# Precision-Engineered Co-N_4−*x*_-C*_x_* Single Atoms Enhance Potential-Resolved Ru(bpy)_3_^2+^ Electrochemiluminescence via Reactive Oxygen Species

**DOI:** 10.34133/research.0842

**Published:** 2025-08-14

**Authors:** Ziqi Kang, Shu Zhu, Shijun Wang, Zhizhi Xiang, Zixin Deng, Xuehao Tong, Zixu Wang, Yanghan Sun, Xiancheng Liu, Guangchao Zang, Chenzhong Li, Guixue Wang, Yuchan Zhang

**Affiliations:** ^1^ Biomedical Innovation and Entrepreneurship Practice Base, Lab Teaching & Management Center, Chongqing Medical University, Chongqing 400016, China.; ^2^ Key Laboratory for Biorheological Science and Technology of Ministry of Education, National Local Joint Engineering Lab for Vascular Implants, College of Bioengineering, Chongqing University, Chongqing 400044, China.; ^3^ JinFeng Laboratory, Chongqing 401329, China.; ^4^ Western Institute of Digital-Intelligent Medicine, Chongqing 401329, China.; ^5^ Bioelectronics and Biosensors Center, School of Medicine, Chinese University of Hong Kong, Shenzhen, Shenzhen 518172, China.; ^6^ College of Biomedical Engineering, Chongqing Medical University, Chongqing 400016, China.

## Abstract

Electrochemiluminescence (ECL) immunoassays based on tris(bipyridine)ruthenium [Ru(bpy)_3_^2+^] is the luminophore representing the most advanced and widely adopted approach in the field of in vitro diagnostics (IVD). However, the scarcity of potential-resolved ECL promoters for Ru(bpy)_3_^2+^ markedly limits its application in clinical diagnostics. Here, we report the first application of cobalt single-atom catalysts (SACs) designed via density functional theory (DFT) calculations to boost the multi-signal ECL of Ru(bpy)_3_^2+^. Mechanism investigations unveil that “ROS accumulation” induced by CoC_4_ and “ROS surge” driven by CoN_4_ are the key factors governing the cathodic and anodic ECL. As a proof of concept, a sandwich ratiometric immunosensor was developed to detect tumor marker CEA and demonstrated excellent clinical feasibility. This work provides insights into the development of tailored ECL promoters by introducing DFT prediction and elucidating the relationships between ORR/HPRR/OER processes and Ru(bpy)_3_^2+^ ECL behavior, paving the way for designing precise immunoassays and advancing IVD techniques.

## Introduction

In vitro diagnostics (IVD), as a convenient method for disease detection, has been widely utilized in clinical medicine. It is estimated that over 2/3 of medical decisions are currently based on IVD results [1]. Among its various branches, immunodiagnostics stands out as the mostimportant [2]. Notably, electrochemiluminescence (ECL) based on tris(bipyridine)ruthenium [Ru(bpy)_3_^2+^] as a luminophore represents the most advanced immunoassay technology mainly due to its excellent potential-resolve capacity [3], currently dominating the majority of the immunodiagnostics market [4]. However, the traditional Ru(bpy)_3_^2+^ detection system suffers from sub-optimal catalytic efficiency of co-reactants, as well as issues of cross-reactivity, which collectively compromise the overall accuracy of the detection [5,6]. Consequently, the induction of potential-resolved luminescence of Ru(bpy)_3_^2+^ and the design of its luminescence enhancers for ratiometric immunoassays have become focal points of research.

Reactive oxygen species (ROS), as intermediates in the oxygen reduction reaction (ORR) and oxygen evolution reaction (OER), have been reported to play an irreplaceable role in the Ru(bpy)_3_^2+^ system, acting as endogenous co-reactants to enhance both cathodic and anodic ECL [7]. However, the restricted regulation of ORR and OER processes results in inadequate ROS accumulation in the detection system, thereby constraining its applicability. Our previous work not only indicates that specific ROS—the superoxide anion (•O_2_H) at −1.70 V and the hydroxyl radical (•OH) at 1.25 V—enhance Ru(bpy)_3_^2+^’s luminescence, but also shows that metal nanoparticle catalysts can regulate ORR/OER to achieve potential-resolved ECL [8]. Yet, efficient strategies have not been developed to precisely design suitable luminescence promoters. Recently, density functional theory (DFT) calculations have been extensively employed to predict molecular structures, electronic configurations, and non-covalent interactions, offering critical insights into structure–property relationships [9–11]. In the context of catalyst design, DFT serves as an effective tool for evaluating energy barriers and charge distribution, thereby facilitating the rational identification and optimization of luminescence promoters with targeted electronic properties [12].

Single-atom catalysts (SACs), renowned for their exceptional efficiency and stability in ORR and OER [13–16], hold important capacity for enhancing the ECL emission of Ru(bpy)_3_^2+^ [17–19]. The atomic-scale structure and coordination of these catalysts can be precisely modulated, facilitating the investigation of the Ru(bpy)_3_^2+^ ECL reaction mechanism and the design of efficient and selective co-reactant accelerators. However, selecting appropriate SACs as promoters is challenging due to the diversity of active metal sites and coordination environments. Moreover, Co atoms were famous for their recognized catalytic excellence in ORR and OER, receiving increasing attention.

In this study, a series of SACs were designed with cobalt as central atom and C/N as coordination atoms. DFT calculation was performed to identify the catalytic capacity. The result shows that CoC_4_ and CoN_4_ selectively catalyzed the incompletely ORR to ROS and the 4e^−^ORR to H_2_O, respectively. Additionally, the CoN_4_ possesses the capability to catalyze both the 2e^−^ORR and HPRR, whereas CoC_4_ exhibits minimal ability in both. Based on DFT calculation results, CoC_4_@graphene and CoN_4_@graphene were synthesized, with electrochemical tests validating our theoretical insights. Furthermore, ROS accumulation theory was first proposed to elucidate the cathodic emission mechanism. Notably, CoN_4_@graphene and CoC_4_@graphene showed promising anodic and cathodic ECL responses with Ru(bpy)_3_^2+^, thanks to the providential catalytic ability, which markedly reduces luminescence crosstalk within the detection system. Leveraging this, a Co-SACs-enhanced ECL ratiometric immunosensor was developed, which shows impressive performance, achieving a detection limit of 10^−15^ g ml^−1^ and exhibiting superior linearity, selectivity, and stability.

**Fig. 1.**
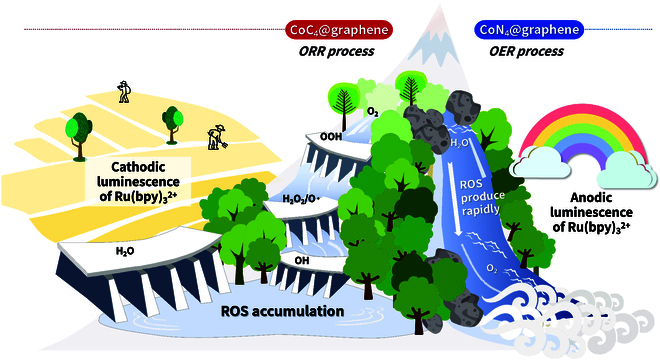
Schematic illustration of the potential-resolved luminescence of Ru(bpy)_3_^2+^ obtained by regulating the ORR/OER catalytic performance of Co SACs. In the cathodic, CoC_4_@graphene exhibits incomplete 3e^−^ORR progression, leading to ROS accumulation. In contrast, the anodic OER driven by CoN_4_@graphene progresses rapidly, triggering a sudden surge of ROS.

This work provides an in-depth analysis of the intrinsic mechanisms by which SACs regulate ORR/OER and their associated coordination changes. We have not only evaluated the capacity of SACs to generate ROS through a 4-electron (4e^−^) ORR, but also considered the impact of 2-electron (2e^−^) ORR and hydrogen peroxide reduction reaction (HPRR) on the luminescence process. The initially proposed “ROS accumulation” pattern further refines the cathodic and anodic excitation mechanisms of ROS-mediated Ru(bpy)_3_^2+^-based ECL systems, offering new perspective and paradigms for the development of Ru(bpy)_3_^2+^ single-atom co-reactant accelerators. Additionally, it enhances the generality and accuracy of ECL sensing analysis in Ru(bpy)_3_^2+^ systems (Fig. 1).

## Results

### Theoretical calculation of ORR performance for cobalt SACs

To investigate the ORR catalytic performance of single-atom Co catalysts under different coordination environments and further understand its impact on the cathodic and anodic luminescence of Ru(bpy)_3_^2+^, we conducted theoretical calculations based on DFT to evaluate the ORR catalytic performance of a series of single-atom Co catalysts. We designed single-atom Co catalysts with different C/N ratios, including CoN_4_, CoC_1_N_3_, CoC_2_N_2_-1, CoC_2_N_2_-2, CoC_3_N_1_, and CoC_4_, as demonstrated in Fig. S1. Notably, CoC_2_N_2_ has 2 different configurations, and the symmetry or asymmetry of the arrangement of C and N atoms may affect the catalytic performance of the Co central atom toward the intermediate process [20]; therefore, CoC_2_N_2_-1 and CoC_2_N_2_-2 were designed.

**Fig. 2.**
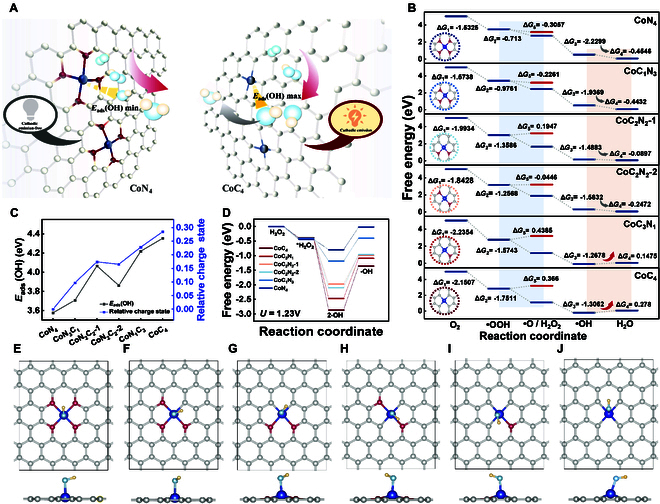
DET calculation. (A) Schematic illustration of theoretical prediction for SACs suitable for the Ru(bpy)32+-ECL system. (B) Calculated free energy diagrams for the ORR at *U* = 0 V. (C) Relative charge states and corresponding OH* adsorption energies of the Co atom of CoC*_x_*N_4−*x*_. (D) Calculated free energy diagrams for the HPRR at *U* = 1.23 V. The optimized structures for the adsorption of •OH on (E) CoN_4_, (F) CoC_1_N_3_, (G) CoC_2_N_2_-1, (H) CoC_2_N_2_-2, (I) CoC_3_N_1_ and (J) CoC_4_. Color scheme: C, gold; N, gray; Co, blue.

Through the analysis of the computational calculations depicted in Fig. 2B and Table S1, the most positive delta *G* (Δ*G*) is observed in the fourth step (*OH + H^+^ + e^−^ → * + H_2_O), indicating that this is the rate-limiting process for the 4e^−^ORR. Moreover, the magnitude of Δ*G*_4_ (Gibbs free energy from •OH to H_2_O in the 4e^−^ORR) for Co SACs with different C/N ratios follows a certain pattern in which the greater the number of carbon atoms involved in coordination is, the larger the Δ*G*_4_ value is, and the poorer the catalytic performance of Co SACs for the ORR is. This phenomenon is consistent with the findings of Zhang et al. [21]. The Δ*G*_4_ values for CoN_4_, CoC_1_N_3_, CoC_2_N_2_-1, and CoC_2_N_2_-2 increase progressively but remain negative (−0.455 eV, −0.443 eV, and −0.090 eV, respectively), implying that all the electron-transfer steps of the ORR are exothermic reactions, allowing for the successful progression of the 4e^−^ORR. However, the positive Δ*G*_4_ values for CoC_4_ and CoC_3_N_1_ (0.278 and 0.147 eV, respectively) indicate that the 4e^−^ORR cannot proceed completely to produce the final product (H_2_O) when *U* = 0 [22]. However, before this step, all electron transfer reactions are exergonic, enabling the generation of ROS such as •OOH, •O, and •OH. Therefore, the ORR process by CoC_4_ and CoC_3_N_1_ leads to the accumulation of ROS in the system. Additionally, the adsorption energy [*E*_ads_(•OH)] and relative charge state of CoC*_x_*N_4−*x*_ for •OH further substantiated the aforementioned conclusion. As shown in Fig. 2C, as the number of C atoms coordinated to single cobalt atoms increases, the *E*_ads_(•OH) becomes larger, and the relative charge states show a consistent trend, revealing greater stability to maintain this state and less likelihood of proceeding to the next step. ORR catalytic performance of CoC*_x_*N_4−*x*_ calculated at *U* = 1.23V shows the same trend (Fig. S2 and Table S2). Additionally, the optimized structures for the adsorption of •OH on CoC*_x_*N_4−*x*_ show that •OH is more inclined to make a vertical connection to the Co sites in CoN_4_, while its connections to the Co sites in CoC_2_N_2_, CoC_3_N_1_, and CoC_4_ are slanted, with a tendency to connect to the Co-C bridge, which is most prominent in CoC_4_, indicating weaker capability for further reaction progression (Fig. 2E to J).

To study the presence of the 2e^−^ORR, we predicted the H_2_O_2_ generation and utilization capabilities of CoC*_x_*N_4−*x*_ species. The step from •OOH to H_2_O_2_ for CoN_4_ and CoC_1_N_3_ is exothermic, indicating their ability to catalyze H_2_O_2_ generation (Fig. 2B). However, for the remaining configurations, this step becomes endothermic with a positive Δ*G*_5_ (Gibbs free energy from •OOH to H_2_O_2_). Among them, CoC_4_ exhibits the highest Δ*G*_5_ value of 0.366 eV, which is greater than its Δ*G*_4_ value of 0.278, suggesting the rare occurrence of 2e^−^ORR and H_2_O_2_ production for CoC_4_ [23].

Theoretical calculations were subsequently conducted to evaluate the HPRR catalytic performance of CoC*_x_*N_4−*x*_. CoN_4_ and CoC_3_N_1_ possess the lowest Δ*G*_6_ values (Gibbs free energy from •OH to H_2_O in the HPRR) of 0.7745 and 0.7869 eV, respectively, indicating higher HPRR catalytic performance (Fig. 2D and Table S3) [24]. This implies that CoN_4_ and CoC_3_N_1_ can generate H_2_O_2_ and effectively catalyze its production of H_2_O, which will avoid background noise in the cathodic portion as an anodic enhancer and is beneficial to detection precision of immunosensors.

### Synthesis and structural characterization of CoC_4_@graphene and CoN_4_@graphene

**Fig. 3.**
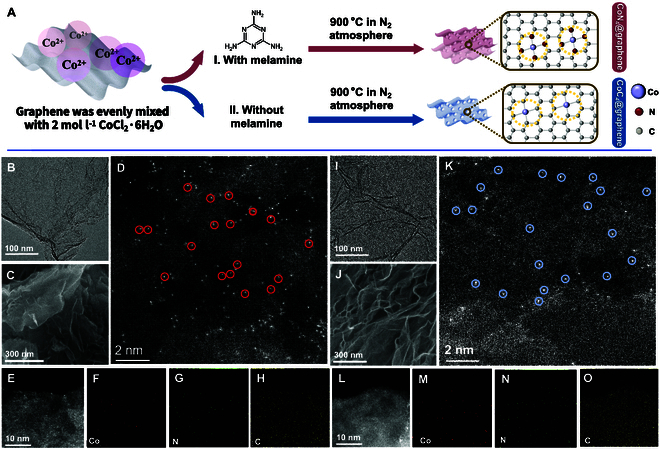
Material synthesis and electron microscopy images. (A) Schematic illustration of the synthesis of Co SACs. (B) TEM, (C) SEM, (D) AC-HAADF-STEM images (isolated bright dots marked with red circles are Co single atoms) and (E to H) corresponding EDS elemental mappings of CoC_4_@graphene. (I) TEM, (J) SEM, (K) AC-HAADF-STEM images (isolated bright dots marked with red circles are Co single atoms), and (L to O) corresponding EDS elemental mappings of CoN_4_@graphene.

**Fig. 4.**
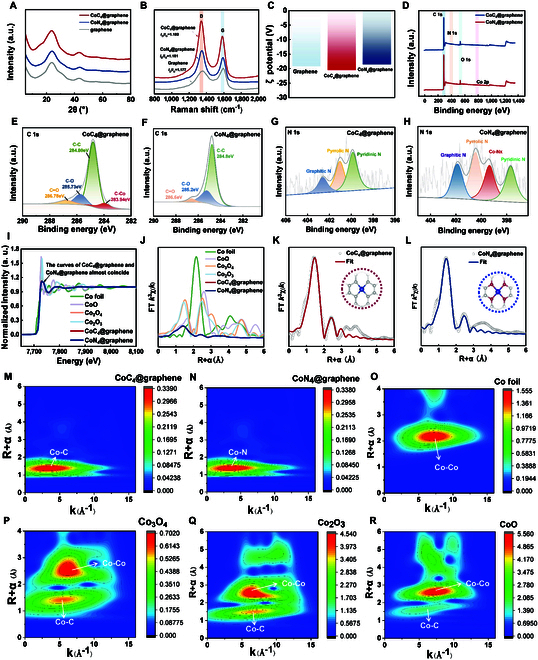
Characterization tests. (A) XRD spectra, (B) Raman spectra, (C) Zeta potential. XPS spectra of (D) overall pattern, C 1s deconvolution for (E) CoC_4_@graphene and (F) CoN_4_@graphene, N 1s deconvolution for (G) CoC_4_@graphene and (H) CoN_4_@graphene. (I) Co K-edge XANES spectra and (J) k^3^-weighted Fourier transform (FT) spectra. Corresponding Co K-edge EXAFS fitting curves of (K) CoC_4_@graphene and (L) CoN_4_@graphene in R space. Wavelet-transformed k^3^-weighted EXAFS spectra of (M) CoC_4_@graphene, (N) CoN_4_@graphene, (O) Co foil, (P) Co_3_O_4_, (Q) Co_2_O_3_, and (R) CoO.

According to the theoretical calculations, we finally chose to synthesize CoC_4_@graphene and CoN_4_@graphene. The catalysts were synthesized via the proposed facile 3-step method, as shown in Fig. 3A [25]. The procedure for sample preparation is detailed in Materials and Methods. Subsequently, several methods were employed to test the successful synthesis of the 2 SACs. Transmission electron microscopy (TEM) images revealed graphene sheet structures in Co SACs, and no nanoparticles were observed (Fig. 3B and I). The scanning electron microscopy (SEM) images of CoN_4_@graphene and CoC_4_@graphene show a hierarchical nanostructure (Fig. 3C and J). Both TEM and SEM images of catalysts are similar to the structure of graphene (Figs. S3 and S4). This phenomenon indicates that both CoC_4_@graphene and CoN_4_@graphene retained the structure of graphene after undergoing a series of processing treatments. Aberration-corrected high-angle annular dark field scanning electron microscopy (AC-HAADF-SEM) images of CoC_4_@graphene and CoN_4_@graphene show atomically distributed Co sites as high-density bright dots, which are highlighted by red circles (CoC_4_@graphene) and blue circles (CoN_4_@graphene) (Fig. 3D and K). The corresponding elemental mappings indicated that Co species are evenly and homogeneously distributed in the carbon skeletons without aggregation in the 2 Co SACs (Fig. 3E to H and L to O). Moreover, the x-ray diffraction (XRD) patterns of CoC_4_@graphene, CoN_4_@graphene, and graphene show no peak at 44.3°, which demonstrates that no large crystals are present in the 2 SAC samples (Fig. 4A) [26]. The broad peak centered at 25.2° can be attributed to the peak of graphitic carbon (002).

Raman spectroscopy was utilized to further elucidate the influence of cobalt atoms on the graphene substrate. As depicted in Fig. 4B, the *I*_D_/*I*_G_ ratio of graphene was measured to be 1.177, whereas the introduction of Co resulted in *I*_D_/*I*_G_ ratios of 1.180 and 1.181 for CoC_4_@graphene and CoN_4_@graphene, respectively. The similarity in the *I*_D_/*I*_G_ ratios of the 3 materials indicates that the impact of cobalt introduction on the graphene substrate is minimal. The Zeta potential of CoN_4_@graphene and CoC_4_@graphene is similar to that of graphene, suggesting that the introduction of Co atoms has minimal impact on the surface characteristics of graphene and excludes the formation of cobalt nanoparticles (Fig. 4C).

We conducted photoelectron spectroscopy (XPS) to measure the coordination of CoC_4_@graphene and CoN_4_@graphene (Fig. 4D). In the C 1 s spectrum of CoC_4_@graphene, there is an obvious peak at 283.94 eV. Considering that no metal elements other than Co participate in the synthesis process, this peak is attributed to the coordination of Co-C (Fig. 4E) [27]. Moreover, this peak does not appear for CoN_4_@graphene and graphene, indicating that there is almost no Co-C coordination in CoN_4_@graphene and graphene (Fig. 4F and Fig. S5A). In CoN_4_@graphene, except for graphene-characteristic pyridine and pyrrolidine nitrogen, a distinct peak appears at 399.2 eV in the N 1 s region, which can be attributed to the formation of Co-N bonds, as reported by Yang et al. [25] (Fig. 4H). Since no Co-N peak was observed in the N 1 spectrum of CoC_4_@graphene and graphene, it can be assumed that Co-N coordination hybrids are rarely formed (Fig. 4G and Fig. S5B). The N contents of the CoC_4_@graphene and CoN_4_@graphene were 2.3 and 3.1 wt%, respectively, as measured by XPS. The N content of CoN_4_@graphene is greater than that of CoC_4_@graphene, but the difference is not very large. In addition, inductively coupled plasma–mass spectrometry (ICP-MS) showed that the content of Co in CoN_4_@graphene (0.27%) was slightly greater than that in CoC_4_@graphene (0.17%), which may be attributed to the greater affinity of N to Co (Table S4). The weak peak intensity in the XPS Co 2p spectra is related to the high dispersion and low content of Co (Fig. S6) [27]. To further investigate the coordination environment and oxidation state of the Co SACs, ToF-SIMS (time of flight secondary ion mass spectrometry) was performed (Fig. S7). A strong Co signal was observed at 58.93 *m*/*z* in both samples, suggesting that Co atoms are present as positively charged ions [28]. Notably, a prominent CoC^+^ fragment was revealed at 70.93 *m*/*z* in CoC_4_@graphene, indicating a stable Co-C coordination environment and a relatively low oxidation state of Co (+1 to +2) [29]. In contrast, CoN_4_@graphene exhibited a stronger CoN^+^ signal at 72.93 *m*/*z*, consistent with predominant Co-N coordination and a Co^2+^ oxidation state [14,30]. These differences underscore the distinct electronic structures and coordination environment of the 2 SACs.

To gain further insight into the atomic coordination environment of CoC_4_@graphene and CoN_4_@graphene, extended x-ray absorption fine structure (EXAFS) measurements and fitting analyses were conducted using synchrotron radiation. The normalized Co K-edge XANES spectra of CoC_4_@graphene and CoN_4_@graphene nearly overlap, suggesting that the Co atoms in both materials possess similar valence states, which are positively shifted relative to metallic Co foil (Fig. 4I). This indicates that cobalt in both materials is in a positively oxidized state. The Fourier transform (FT) of the k^3^-weighted EXAFS spectra shows a primary peak at approximately 1.73 Å for both samples, corresponding to the Co-C and Co-N paths (Fig. 4K and L) [25,31]. Notably, no prominent peak is observed around 2.18 Å, which would be indicative of Co-Co scattering, thereby confirming the atomic dispersion of Co centers without the formation of metallic nanoparticles (Fig. 4J).

EXAFS fitting results reveal that CoC₄@graphene features a Co-C coordination number of 3.5 ± 0.6 with a bond length of 1.99 Å, while CoN₄@graphene exhibits a Co-N coordination number of 4.5 ± 0.7 at 1.94 Å, demonstrating that both have 4-coordinate structures (Table. S5). In both samples, second-shell scattering paths (Co-C-C and Co-N-C, respectively) are observed at approximately 2.86 Å, further supporting the presence of an ordered local coordination environment embedded within the graphene framework [32]. Importantly, no Co-O or Co-Co contributions were detected, excluding the presence of cobalt oxides or clusters. The quality of the fittings is supported by low *R*-factors of 0.0120 for CoC_4_@graphene and 0.0063 for CoN_4_@graphene.

Wavelet transform (WT) analysis was conducted to provide simultaneous resolution in R-space and k-space. As shown in Fig. 4M and N, both CoC_4_@graphene and CoN_4_@graphene display a single intensity maximum centered at ~4 Å^−1^ and 1.4 Å radial distance, which corresponds to Co-C and Co-N light-element coordination paths [33]. In contrast, the WT maps of Co foil and Co-based oxides exhibit dominant signals at ~8 to 12 Å^−1^ and higher R values, indicating heavy-element Co-Co coordination (Fig. 4O to R) [27,34]. Notably, the absence of Co-Co contributions in our samples rules out the formation of Co clusters or oxide phases, which is consistent with the TEM and XPS results. Based on the aforementioned characterizations, we confirmed the successful synthesis of CoN_4_@graphene and CoC_4_@graphene.

### Electrochemical testing of the ORR and HPRR performance of the Co SACs

**Fig. 5.**
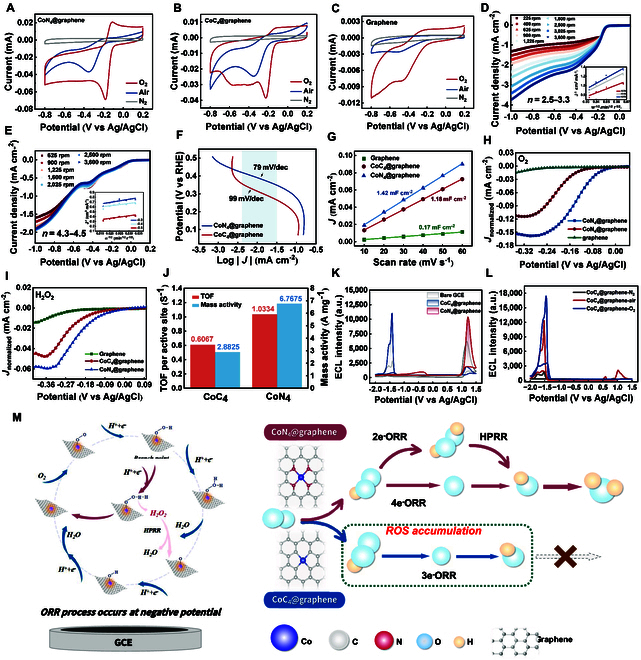
Electrochemical tests. Comparison of the CV curves of (A) CoN_4_@graphene, (B) CoC_4_@graphene, and (C) graphene in different atmospheres in 0.1 M KOH. LSV curves of (D) CoC_4_@graphene and (E) CoN_4_@graphene in O_2_-saturated 0.1 M KOH at various rotation rates (ω) ranging from 225 to 3,600 rpm. The inset shows the K−L plots derived from the RDE measurements. (F) Tafel curves of the proposed Co SACs. (G) *C*_dl_ plot of catalysts. (H) ECSA-normalized LSV curves of RDE at 1,600 rpm in O_2_-saturated 0.1 M KOH. (I) ECSA-normalized LSV curves of RDE at 1,600 rpm in N_2_-saturated 0.1 M KOH and 5 mM H_2_O_2_. (J) TOF and MA of Co SACs. (K) ECL lines of bare GCE (gray), CoC_4_@graphene (blue) and CoN_4_@graphene (red) in 1 mM Ru(bpy)32+ solution. (L) ECL curves of CoC_4_@graphene in different atmospheres in a 1 mM Ru(bpy)32+ solution (−2 to 1.5 V versus Ag wire, scan rate of 0.1 V s^−1^). (M) Schematic illustration of the ORR and HPRR catalytic pathway of CoC_4_@graphene and CoN_4_@graphene.

A series of electrochemical experiments were conducted to validate the computational calculations of the ORR. Cyclic voltammetry (CV) was carried out in 0.1 M KOH using Ag/AgCl as the reference electrode, with the potential swept from 0.2 to –0.8 V under different gas atmospheres. As shown in Fig. 5A and B, both CoC_4_@graphene and CoN_4_@graphene displayed clear cathodic peaks under ambient air, with onset and peak potentials at approximately –0.2 and –0.35 V, respectively. These peaks became more prominent and shifted positively in O₂-saturated conditions, indicating active oxygen involvement and the occurrence of ORR. In contrast, graphene exhibited only a weak response under both conditions, confirming that the ORR activity primarily arises from the dispersed Co single atoms (Fig. 5C). To compare the ORR catalytic activity of each catalyst, linear sweep voltammetry (LSV) measurements were conducted under O₂-saturated 0.1 M KOH using a rotating disk electrode (RDE) at 1,600 rpm. As shown in Fig. S10, CoN₄@graphene displayed a more positive onset potential (0.01 V vs. Ag/AgCl) and a higher half-wave potential (*E*_1/2_ = −0.13 V vs. Ag/AgCl) compared to CoC₄@graphene (onset potential –0.12 V vs. Ag/AgCl, *E*_1/2_ = −0.2 V vs. Ag/AgCl). LSV measurements on CoC_4_@graphene and CoN_4_@graphene were conducted at RDE at rotation speeds ranging from 225 to 3,600 rpm to further elucidate the catalytic performance. The number of transferred electrons (*n*) per oxygen molecule (O_2_) for CoC_4_@graphene is 2.5 to 3.3, and that for CoN_4_@graphene is 4.3 to 4.5 according to the Koutecky–Levich (K–L) equation (Fig. 5D and E), which is in agreement with the results of the computational calculations. The rotating ring-disk electrode (RRDE) measurement was also employed as a complementary method to support the results obtained from RDE (Fig. S11).

The catalytic efficiency of CoN_4_@graphene and CoC_4_@graphene was further demonstrated by the Tafel curve, an intuitive criterion for discerning the activity of electrocatalysts. The Tafel slope of CoN₄@graphene (79 mV dec^−1^) was notably lower than that of CoC₄@graphene (99 mV dec^−1^), reflecting more favorable ORR kinetics and reduced overpotential (Fig. 5F).

The influence of H_2_O_2_-related reaction especially HRPP was also taken into consideration. As shown in Fig. S12, LSV conducted in 0.1 M KOH containing 5 mM H₂O₂ revealed that CoN₄@graphene exhibits an earlier onset potential (−0.09 V vs. Ag/AgCl) and a more positive half-wave potential (−0.23 V) compared to CoC₄@graphene (−0.17 and −0.26 V, respectively). These results demonstrate the superior HPRR activity of CoN₄@graphene, in agreement with theoretical predictions [35].

To further assess the intrinsic catalytic activity, the electrochemically active surface area (ECSA) was estimated based on the double-layer capacitance (*C*_dl_) extracted from non-Faradaic CV measurements (Fig. S13) [36]. As shown in Fig. 5G, CoN₄@graphene exhibited the highest *C*_dl_ (1.42 mF·cm^−2^), followed by CoC₄@graphene (1.18 mF·cm^−2^) and graphene (0.17 mF·cm^−2^), suggesting a higher density of electrochemically accessible sites for CoN₄@graphene. Subsequently, LSV curves were normalized by ECSA to better reflect the intrinsic activity. The ORR normalized diffusion-limited current densities were –0.155 and –0.118 mA cm^−2^ for CoN₄@graphene and CoC₄@graphene, respectively, indicating the superior intrinsic catalytic performance of CoN₄@graphene (Fig. 5H). A consistent trend was observed in HPRR, where CoN₄@graphene demonstrated a higher normalized diffusion-limited current density (–0.060 mA·cm^−2^) than CoC₄@graphene (–0.047 mA·cm^−2^), accompanied by a more positive onset potential (Fig. 5I). These findings collectively validate the superior intrinsic activity of CoN₄@graphene in both ORR and HPRR. The turnover frequency (TOF) and mass activity (MA) were calculated at a representative overpotential of 300 mV. As shown in Fig. 5J, CoN_4_@graphene exhibited a markedly higher TOF of 1.0334 s^−1^, nearly 1.7 times that of CoC_4_@graphene (0.6067 s^−1^), indicating superior per-site catalytic activity. Meanwhile, the MA of CoN_4_@graphene reached 6.7675 A mg^−1^, far exceeding the 2.8825 A mg^−1^ achieved by CoC_4_@graphene. This enhancement demonstrates not only the improved intrinsic activity but also the more efficient utilization of active Co sites in the nitrogen-coordinated configuration, which is in good agreement with theoretical predictions and previous electrochemical analyses [37].

As schematically demonstrated in Fig. 5M, our electrochemical experiments confirmed the catalytic capability of CoN_4_@graphene and CoC_4_@graphene in oxygen-related reduction reactions. CoN_4_@graphene exhibits robust 4e^−^ORR catalysis along with a 2e^−^ORR pathway and H_2_O_2_ reduction, ultimately resulting in the formation of the final product H_2_O. In contrast, CoC_4_@graphene demonstrates 3e^−^ORR catalysis without a 2e^−^ORR pathway, leading to the accumulation of ROS.

Subsequently, 5-μl solutions of CoC_4_@graphene and CoN_4_@graphene were drop-cast onto the surface of glassy carbon electrodes (GCEs) for testing in a 0.1 M Ru(bpy)_3_^2+^ solution at pH 6 with cyclic voltages ranging from −2 to 1.5 V vs. Ag/AgCl. Satisfactory cathodic ECL emission was boosted by CoC_4_@graphene at −1.5 V with an intensity of 11,021 a.u. (Fig. 5K). A simple experiment was conducted to verify the participation of O_2_ in the ECL system. Under oxygen-saturated conditions, the cathodic luminescence boosted by CoC_4_@graphene markedly increased to 17,402 a.u., whereas it was noticeably suppressed under nitrogen-saturated conditions (Fig. 5L). The results confirm the crucial role of oxygen and the ORR in the luminescence of Ru(bpy)_3_^2+^ at −1.5 V. Conversely, when CoN_4_@graphene was employed in the same system, cathodic ECL intensity was barely influenced by the O_2_ (Fig. S14).

### Possible mechanism of the anodic luminescence of Ru(bpy)_3_^2+^ promoted by CoN_4_@graphene

**Fig. 6.**
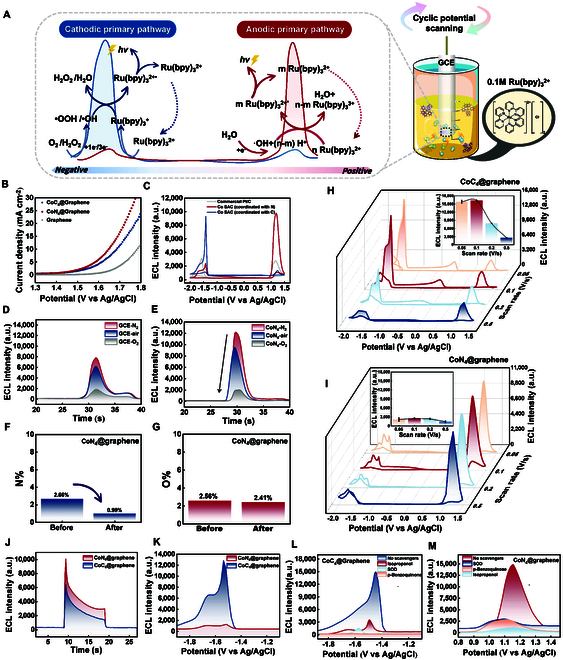
Mechanism investigations. (A) Schematic illustration of the potential-resolved ECL mechanism of Ru(bpy)_3_^2+^. (B) Polarization curves of CoC_4_@graphene, CoN_4_@graphene, and graphene. (C) ECL curves of CoC_4_@graphene, CoN_4_@graphene, and commercial Pt/C. ECL curves of (D) bare GCE and (E) CoN_4_@graphene in different atmospheres. Ratio of (F) nitrogen atoms and (G) oxygen atoms to the total amount of carbon, oxygen, and nitrogen atoms of CoN_4_@graphene before and after a 1-h reaction with 1 mM Ru(bpy)_3_^2+^ (measured by XPS). ECL curves of (H) CoC_4_@graphene and (I) CoC_4_@graphene in a 1 mM Ru(bpy)_3_^2+^ solution at scan rates of 0.1 V s^−1^ (red), 0.05 V s^−1^ (orange), 0.2 V s^−1^ (light blue), and 0.5 V s^−1^ (dark blue); inset: the corresponding cathodic ECLs at different scan rates. The Ru(bpy)_3_^2+^ cathodic luminescence of (J) time-dependent ECL and (K) cyclic voltammetry ECL of CoC_4_@graphene (blue line) and CoN_4_@graphene (red line). ECL responses of (L) CoC_4_@Graphene and (M) CoN_4_@Graphene in 0.1 M Ru(bpy)_3_^2+^ containing 0.05 mM benzoquinone, 0.05 mM SOD, 0.05 mM isopropanol, and control group.

Multiple electrochemical experiments were carried out to clarify the anodic luminescence mechanism, which was finally concluded as pathway 1 and pathway 2 (Fig. S15, pink). We assume that the mechanism of the anodic ECL of Ru(bpy)_3_^2+^ is related to the precise OER catalysis by CoN_4_@graphene. According to the electrochemical experiments (Fig. 6B), CoN_4_@graphene and CoC_4_@graphene catalyze the OER at approximately 1.30 and 1.34 V, respectively (H_2_O − e^−^ → •OH + H^+^). Correspondingly, the oxidation potential of Ru(bpy)_3_^2+^ was approximately 1.13 V, indicating that Ru(bpy)_3_^2+^ began to be oxidized and that Ru(bpy)_3_^3+^ was formed at this potential [Ru(bpy)_3_^2+^ − e^−^ → Ru(bpy)_3_^3+^] [38]. Consequently, since the oxidation potential of Ru(bpy)_3_^2+^ precedes the potential at which Co SACs initiate OER catalysis, the accumulation of Ru(bpy)_3_^3+^ in the system can quickly utilize the surged ROS (mainly •OH, which is the product of the first step of the OER) generated by the CoN_4_-catalyzed OER. Subsequently, Ru(bpy)_3_^3+^ reacts with •OH to generate excited-state Ru(bpy)_3_^2+^ [Ru(bpy)_3_^2+*^], which, upon returning to the ground state, emits photons. For the weaker OER catalytic CoC_4_@graphene, the OER onset potential of 1.34 V occurred slightly later than that of CoN_4_@graphene, and its weak OER catalytic performance was insufficient to generate enough ROS for the utilization of Ru(bpy)_3_^3+^. Therefore, it does not promote the anodic emission of Ru(bpy)_3_^3+^. We also employed the widely recognized excellent commercial Pt/C OER catalyst for electrochemical and enhanced chemiluminescence (ECL) testing. The results revealed that the catalytic OER ability of the Pt/C catalyst was greater than that of CoN_4_@graphene [39], but its ability to promote Ru(bpy)_3_^2+^ anodic ECL was only approximately 3,000 a.u. (Fig. 6C). This can be attributed to the excessively strong OER catalytic performance leading to the overgeneration of the final product O_2_, which quenches the anodic luminescence of Ru(bpy)_3_^2+^ [40].

Hence, to further substantiate the existence of an O_2_ quenching effect, we performed an ECL test using a bare GCE and CoN_4_@graphene in a 0.1 M Ru(bpy)_3_^2+^ system in atmospheres of O_2_, air, and N_2_ (Fig. 6D). As the oxygen concentration increases in the atmosphere, ECL intensity diminishes. In the presence of CoN_4_@graphene, the anodic emission of Ru(bpy)_3_^2+^ was notably suppressed to approximately 2,018 a.u. in an O_2_ atmosphere, which was much lower than the 10,100 a.u. in air. On the other hand, in an N_2_ atmosphere, the emission increased to 12,300 a.u. (Fig. 6E). Thus, the inhibitory effect of O_2_ on the anodic luminescence of Ru(bpy)_3_^2+^ persisted regardless of the presence or absence of an ECL promoter.

We elucidated the mechanism of the ECL emission of Ru(bpy)_3_^2+^ at the anode. First, H_2_O undergoes an appropriate OER catalyzed by CoN_4_@graphene, generating •OH. Due to its powerful oxidizing ability, OH excites a fraction of Ru(bpy)_3_^2+^ to form Ru(bpy)_3_^2+*^, which then returns to the ground-state emitting photon.

Moreover, the coreactant pathway was taken into account. XPS analysis was performed on CoN_4_@graphene before and after a 2-h reaction with Ru(bpy)_3_^2+^ to confirm the nitrogen content, revealing a decrease from 2.66% to 0.99% (Fig. 6F). The change in oxygen content before and after the reaction was minimal (Fig. 6G). We hypothesize that a small amount of -NH_2_ groups on the surface of CoN_4_@graphene promote Ru(bpy)_3_^2+^ to an excited state, which is similar to tripropylamine (TrA) [38,41,42]. Under an anodic voltage, both Ru(bpy)_3_^2+^ and a small amount of CoN_4_@graphene undergo oxidation reactions at the electrode surface, yielding Ru(bpy)_3_^3+^ and CoN_4_@graphene•, respectively. Subsequently, CoN_4_@graphene• undergoes a deprotonation process, generating a strongly reducing intermediate (CoN_4_@graphene•), which reacts with Ru(bpy)_3_^3+^ to form excited Ru(bpy)_3_^2+*^, leading to ECL emission.

The above findings were summarized by the following equations (Eqs. 1 to 8):

$$H_{2}O-e^{-}\to •\mathrm{OH}+H^{+}$$ (1)

Pathway 1 (primary):

nRubpy32++•OH+n−mH+→mRubpy32+∗+n−mRubpy33++mH2O (2)

mRubpy32+∗→mRubpy32++hv (3)

Pathway 2:

CoN4@grapheneArNRCH2R′−e−→CoN4@grapheneArNRCH2R′+• (4)

CoN4@grapheneArNRCH2R′+•−H+→CoN4@grapheneArNRCHR′• (5)

CoN4@grapheneArNRCHR′•+Rubpy33+→CoN4@grapheneArNR=CHR′++Rubpy32+∗ (6)

CoN4@grapheneArNR=CHR′++H2O−H+→CoN4@grapheneArNRH•+R′CHO (7)

Rubpy32+∗→Rubpy32++hv (8)

### Possible mechanism of the cathodic luminescence of Ru(bpy)_3_^2+^ promoted by CoC_4_@graphene

To confirm the detailed dynamics in negative potential, a set of investigation was carried out. Pathway 3 and pathway 4 show the detailed process of the cathodic emission (Fig. S15, blue). Building upon the previous results, CoN_4_@graphene and CoC_4_@graphene initiated the ORR catalytic process at approximately 0 and −0.2 V, respectively. Moreover, CoN_4_@graphene catalyzes the 4e^−^ORR, while CoC_4_@graphene facilitates the 3e^−^ORR. Specifically, CoN_4_@graphene can catalyze the complete ORR to produce the final product H_2_O, whereas the ORR catalyzed by CoC_4_@graphene can successfully complete the first 3 steps of electron transfer but cannot complete the final step of electron transfer (•OH + H^+^ + e^−^ → H_2_O). This is consistent with the theoretical results from DFT calculations. Therefore, we believe that the variation in the ability of Co SACs to enhance cathodic luminescence mainly lies in the extent of the chemical processes they catalyze and the resulting accumulation of ROS in the system. Ru(bpy)_3_^2+^ begins to be reduced at approximately −1.60 V [Ru(bpy)_3_^2+^ + e^−^ → Ru(bpy)_3_^3+^] [38]. Therefore, there is a certain distance from the initiation of Co SACs catalyzing the ORR to the formation of Ru(bpy) _3_^3+^ at −1.60 V. Thus, the effective accumulation of ROS from low potentials to −1.6 V during potential scanning is crucial for exciting the Ru(bpy)_3_^2+^ cathodic ECL. Since the 4e^−^ORR reaction catalyzed by CoN_4_@graphene is rapid and complete, the final H_2_O product is directly obtained; thus, the final H_2_O product fails to accumulate ROS in the system, while CoC_4_@graphene regulates the incomplete 3e^−^ORR reaction, which is favorable for the accumulation of ROS in the system. These accumulated ROS contribute to promoting the cathodic luminescence of Ru(bpy)_3_^2+^.

The “ROS accumulation” theory was subsequently validated by investigating the changes in the cathodic ECL intensity as a function of the scan rate. Increasing the scan rate results in a decrease in free radical accumulation, while decreasing the scan rate leads to an increase in free radical accumulation due to the prolonged reaction time [43]. As depicted in Fig. 6H, when the scan rate was 0.5 V s^−1^, the CoC_4_-boosted cathodic ECL of Ru(bpy)_3_^2+^ decreased to only 2,592 a.u. When the scan rate decreased to 0.2 V s^−1^, the ECL intensity increased to 7,904 a.u., while when the scan rate decreased to 0.1 V s^−1^, the ECL intensity increased to 16,280 a.u. Further decreases in the scan rate did not cause obvious changes in the ECL intensity. This can be attributed to the equilibrium reached between the accumulation and consumption of ROS. This experiment not only demonstrated the occurrence of the free radical accumulation process but also confirmed that 0.1 V s^−1^ was the most optimized scanning rate. For CoN_4_@graphene, the scan rate had no obvious influence on the cathodic ECL, indicating a deficiency in ROS accumulation (Fig. 6I). The proposed “ROS accumulation” theory was also demonstrated by other similar study. In a recent study, Zhou et al. [44] encapsulated luminol within the framework of ZIF-8@AuNPs, enabling the accelerated generation and rapid accumulation of superoxide anions and hydroxyl radicals within the confined nanoenvironment, thereby facilitating immediate reaction with luminol anion radicals. This finding provides strong experimental support for the “ROS accumulation” mechanism.

Thereafter, time-dependent ECL (Td ECL) was employed to investigate the influence of Co SACs on the cathodic ECL of Ru(bpy)_3_^2+^ by eliminating the process of free radical accumulation. *E*_0_ = 0 V was applied for 10 s, followed by a step to *E*_red_ = −1.6 V for another 10 s, and the aforementioned voltage switching cycle was repeated. As *E*_red_ was applied, the ECL emission rapidly reached its maximum peak and subsequently exhibited a logarithmic decline, which indicates a logarithmic decrease in the concentration of ROS on the electrode surface (Fig. 6J). This phenomenon is consistent with the conclusion reported by Wu et al. [45]. The Td ECL results revealed that CoC_4_@graphene promoted the cathodic ECL of Ru(bpy)_3_^2+^ by approximately 6,326 a.u., which is lower than the 13,280 a.u. observed in the CV measurement, where ROS accumulation occurs (Fig. 6K). In contrast, due to its stronger ORR catalytic performance, CoN_4_@graphene can enhance the cathodic ECL of Ru(bpy)_3_^2+^ by approximately 10,000 a.u. This suggests that when the potential is switched to −1.6 V, where Ru(bpy)_3_^2+^ is reduced, both CoC_4_@graphene and CoN_4_@graphene promote the ORR to produce ROS, which can be directly and rapidly utilized by Ru(bpy)_3_^3+^. Thus, the cathodic ECL of Ru(bpy)_3_^2+^ depends solely on the ORR efficiency and is unaffected by ROS accumulation. Hence, the ROS accumulation process is proven to be crucial to cathodic luminescence of Ru(bpy)_3_^2+^. The outstanding cathodic ECL performance of CoC_4_@graphene was confirmed by comparing with other cathodic ECL enhancers (Fig. S16).

To further investigate the contributions of different types of ROS to the anodic and cathodic luminescence of Ru(bpy)_3_^2+^, ROS scavengers were introduced into the system and observed changes in the ECL intensity (Fig. 6L). The cathodic ECL emission of Ru(bpy)_3_^2+^ apparently decreased in the presence of ROS scavengers. However, overall, the decrease was greater when •OOH scavengers were added than when •OH scavengers were added. In the case of the anodic ECL promoted by CoN_4_@graphene, the attenuations caused by •OH scavengers were greater than those caused by •OOH scavengers (Fig. 6M). These observations suggest that ROS contribute to both cathodic and anodic ECL of Ru(bpy)_3_^2+^, with •OOH and •OH playing dominant roles in promoting cathodic and anodic ECL, respectively.

We provided a detailed elucidation of the cathodoluminescence mechanism. ROS are generated and accumulate in the system. Ru(bpy)_3_^2+^ receives an electron, resulting in the formation of Ru(bpy)_3_^+^, which subsequently reacts with ROS to generate Ru(bpy)_3_^2+*^, and ECL emission occurs. Alternatively, due to the strong oxidation capability of •OH, Ru(bpy)_3_^2+^ is oxidized to Ru(bpy)_3_^3+^, which reacts with Ru(bpy)_3_^+^ to regenerate Ru(bpy)_3_^2+*^.

The cathodoluminescence mechanism of Ru(bpy)_3_^2+^ was concluded with the following equations (Eqs. 9 to 17):

$$O_{2}+e^{-}+H^{+}\to •O_{2}H$$ (9)

$$O_{2}+3e^{-}+H^{+}\to 3•\mathrm{OH}$$ (10)

$$\;\mathrm{Ru}{{\left(\mathrm{bpy}\right)}_{3}}^{2+}+e^{-}\to \mathrm{Ru}{{\left(\mathrm{bpy}\right)}_{3}}^{+}$$ (11)

Pathway 3:

$$\;\mathrm{Ru}{{\left(\mathrm{bpy}\right)}_{3}}^{+}+•O_{2}H+H^{+}\to \mathrm{Ru}{{\left(\mathrm{bpy}\right)}_{3}}^{2+*}+H_{2}O_{2}$$ (12)

$$\;\mathrm{Ru}{{\left(\mathrm{bpy}\right)}_{3}}^{+}+•\mathrm{OH}+H^{+}\to \mathrm{Ru}{{\left(\mathrm{bpy}\right)}_{3}}^{2+*}+H_{2}O$$ (13)

$$\;\mathrm{Ru}{{\left(\mathrm{bpy}\right)}_{3}}^{2+*}\to \mathrm{Ru}{{\left(\mathrm{bpy}\right)}_{3}}^{2+}+\mathrm{hv}$$ (14)

Pathway 4:

$$\;\mathrm{Ru}{{\left(\mathrm{bpy}\right)}_{3}}^{2+}+•\mathrm{OH}+H^{+}\to \mathrm{Ru}{{\left(\mathrm{bpy}\right)}_{3}}^{3+}+H_{2}O$$ (15)

$$\;\mathrm{Ru}{{\left(\mathrm{bpy}\right)}_{3}}^{+}+\mathrm{Ru}{{\left(\mathrm{bpy}\right)}_{3}}^{3+}\to \mathrm{Ru}{{\left(\mathrm{bpy}\right)}_{3}}^{2+*}+\mathrm{Ru}{{\left(\mathrm{bpy}\right)}_{3}}^{2+}$$ (16)

$$\;\mathrm{Ru}{{\left(\mathrm{bpy}\right)}_{3}}^{2+*}\to \mathrm{Ru}{{\left(\mathrm{bpy}\right)}_{3}}^{2+}+\mathrm{hv}$$ (17)

### Construction, characterization, and application of Co SAC-based ratiometric immunosensors

**Fig. 7.**
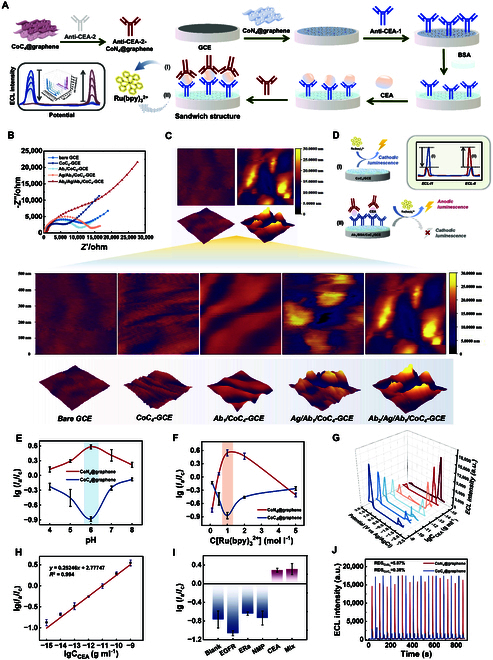
Construction, characterization, and application of the immunosensor. (A) Schematic illustration of the construction process of the proposed immunosensor. (B) EIS image and (C) AFM images of the proposed immunosensor. EIS was measured in 0.1 mol l^−1^ KCl containing 5.0 mmol l^−1^ K_3_[Fe(CN)_6_]/K_4_[Fe(CN)_6_] (1:1). (D) Schematic illustration of the detection principle of the proposed sensor. (E) Optimization of the pH and (F) concentration of Ru(bpy)_3_^2+^ in the sensor. (G) ECL responses and (H) calibration plot of the ratiometric biosensor with different concentrations of CEA ranging from 10^−7^ to 10^−1^ ng ml^−1^ in 1 mM Ru(bpy)_3_^2+^. (I) Selectivity and (J) stability of the proposed ratiometric ECL biosensor.

A ratiometric immunosensor was built utilizing the proposed potential-modulated ECL-promoting SACs to verify its clinical detection potential. CoC_4_@graphene and CoN_4_@graphene were respectively drop-cast onto the electrode surface and loaded as labels on the secondary antibody (Ab_2_) to assemble a sandwich-type immunosensor for the detection of trace biomarkers in body fluids, with carcinoembryonic antigen (CEA) as a representative example. Figure 7A illustrates the fabrication process of the immunosensor.

Electrochemical impedance spectroscopy (EIS) was performed using a 3-electrode system in a 5 mM [Fe(CN)_6_]^3−/4−^ solution to demonstrate the successful fabrication of the immunosensor. As depicted in Fig. 7B, the CoC_4_-GCE layer had a smaller radius of curvature than did the bare electrode, which was attributed to the excellent conductivity of graphene. Upon the addition of Ab_1_, Ag (CEA), and Ab_2_, the radius increased due to increased protein resistance, confirming successful immunosensor construction [46–49]. Atomic force microscopy (AFM) was employed to provide further visual evidence. The surface thickness clearly increased after the sensor was built (Fig. 7C). This result confirmed the successful establishment of the biosensor. The relationship of ECL intensity change and CEA concentration is schematically shown in Fig. 7D. To determine the optimal testing conditions for the sensor, we optimized the pH and concentration of the Ru(bpy)_3_^2+^ complex electrolyte (Fig. 7E and F). A pH of 6 and a Ru(bpy)_3_^2+^ concentration of 1 mM was ultimately selected for the detection system.

The constructed immunosensor was used to detect different concentrations of CEA under the optimal detection conditions. As shown in Fig. 7G, the cathodic ECL signal decreased, and the anodic ECL signal increased correspondingly with increasing CEA concentration. Moreover, Ig (*I*_a_/*I*_c_) shows a satisfactory relationship with the logarithm of the CEA concentration from 10^−15^ to 10^−9^ ng ml^−1^. The corresponding linear equation was *y* = 0.25246*x* + 2.77747, *R*^2^ = 0.994; the limit of detection (LOD) was estimated to be 33 fg ml^−1^ (Fig. 7H).

Subsequently, some important properties of biosensors were explored. We evaluated the selectivity of this system by choosing epidermal growth factor receptor (EGFR) (10^−2^ ng ml^−1^), estrogen receptor alpha (ERα) (10^−2^ ng ml^−1^), and nuclear matrix protein 22 (NMP22) (10^−2^ ng ml^−1^) as the interfering substances, all of which are common interfering biomolecules existing in the blood of both patients and normal human. As depicted in Fig. 7I, the ECL intensity of GFR, ERα, and NMP22 was similar to that of the blank solution, while a high ECL response was obtained in the presence of CEA. Furthermore, the ECL intensity of the mixed solution was similar to that of CEA alone. The results indicate that the system has good selectivity for CEA. The stability of the immunosensor was also explored under consecutive scans from −1.5 to 2 V vs. Ag/AgCl for 15 cycles. Both the cathodic and anodic luminescence remained considerably stable, with relative standard deviations of 5.87% and 0.38%, respectively (Fig. 7J).

To further evaluate the clinical detection and analysis capabilities of this immunosensor, real samples were subjected to testing. Serum specimens from 3 healthy individuals were collected. Using a spiked method, known concentrations (1×10^−12^ g ml^−1^) of CEA solution were added to the samples, which were then analyzed using the developed immunosensor. The Ig (*I*_a_/*I*_c_) values were recorded, and the CEA concentrations were determined by a standard curve. Each sample was tested 3 times. The means of 3 repeated tests and the RSDs are presented in Table S6. The final results indicate that no obvious difference was present between the detected CEA and added CEA, with the RSD fluctuating between 5.53% and 8.71%. Therefore, we believe that this sensor holds great potential for clinical applications.

## Discussion

In this work, cobalt SACs with different coordination were elaborately designed using DFT calculation to predict a series of oxygen-related reaction. Apparently, CoC_4_@graphene demonstrated notable 3e^−^ORR catalytic activity, ensuring the accumulation of ROS in the system and thereby promoting excellent cathodic luminescence. Conversely, CoN_4_@graphene exhibited superior anodic luminescence owing to its appropriate OER catalytic performance and the presence of NH_2_ groups on its surface. Finally, a ratiometric CEA immunosensor with perfect detection capacity was designed.

Beyond tumor marker detection, our findings also have great potential in in vivo and in vitro bioassay and may provide insight in future sensor designs. We employed materials with good bioaffinity. A graphene-based substrate with favorable biocompatibility was used [50], and the introduction of single-atom catalytic sites, which avoids the biological toxicity associated with nanoparticles [51], thereby providing conditions for in situ tissue/cell detection [52].

However, current real-time cellular detection is still mostly limited to small molecules such as dopamine. The scarcity of single-luminescence-with-dual-emission systems may be a major reason for the limited development of biomacromolecule sensing, as achieving dual luminescence without introducing additional small molecules imposes extremely high requirements on the sensing system itself [8]. Our work provides a solution to this challenge, filling the gap in biomacromolecule detection and promoting advances in immunoassay development.

Meanwhile, the 2 luminescence promoters employed differ only in the coordination environment of the central atom, which eliminates systematic errors caused by differences between materials during the detection process. The excellent stability of SACs also offers a potential solution to the complex environments encountered in in vivo detection [53]. In addition, the oxygen-related catalytic capability of SACs opens new possibilities for oxidative stress detection and physiological function regulation.

There are still some limitations in our study that can be improved in the future biosensing research. Although SACs offer precise catalytic control, facile tunability, and well-established computational modeling capabilities, they still face challenges such as synthetic difficulty and stringent equipment requirements [54]. Typically, SAC synthesis demands high temperatures, which may damage oxygen-containing functional groups anchored on the substrate and consequently reduce water solubility [55]. In contrast, nanoparticles are easier and faster to synthesize with minimal equipment requirements. By focusing on specific crystallographic facets of nanoparticles for computational modeling, it becomes possible to predict and design optimal catalytic configurations for luminescence enhancement, thus enabling directed design and precise catalysis [56]. Such nanoparticle-based systems hold considerable potential for IVD.

Currently, dual-channel ECL platforms based on the potential-resolved system are primarily utilized either for ratiometric sensing or for multi-analyte detection [7]. However, the simultaneous optimization of these 2 capabilities remains difficult, owing to intrinsic design constraints of the system [57]. Without introducing additional emitters, such platforms can only achieve accurate detection by ratio strategy for a single biomarker. Conversely, when adapted for multi-marker detection, they often compromise precision. To realize accurate and simultaneous multi-biomarker detection, more sophisticated system architectures are required. The synergistic integration of potential-resolved and spectrally resolved strategies introduces an additional dimension of analytical control, thereby enabling both high diagnostic accuracy and quantitative precision. Such advancements could pave the way for next-generation diagnostic systems capable of high-throughput and multiplexed biomarker detection. However, realizing such systems in practice requires overcoming substantial challenges, particularly in selecting and designing luminophores, minimizing potential overlap, and resolving accurate spectral signal. Continued innovation in ECL system design, luminophore engineering and co-reactants optimization will be crucial to fully unlock the potential of multi-dimensional platforms in clinical diagnostics.

While our work focuses on employing DFT calculations to design and screen co-reactant accelerators for enhancing Ru(bpy)₃^2+^-based ECL, the application of DFT in developing novel luminophores themselves holds even greater potential. DFT has proven to be a powerful tool for elucidating structure–function relationships by providing insight into energy levels, charge distribution, and excited-state behavior [58–60]. To date, most DFT-driven studies of luminescent mechanisms have centered on covalent-organic frameworks (COF)- and metal-organic frameworks (MOF)-based luminophores, whose periodic crystalline frameworks facilitate precise electronic modeling [61,62]. In contrast, traditional luminophores such as Ru(bpy)₃^2+^ and luminol remain challenging for DFT due to their conformational flexibility and solvent-dependent excited-state dynamics [63,64]. To address these limitations, researchers have incorporated Ru complexes into MOF/COF scaffolds, thereby enhancing their ECL performance while enabling reliable theoretical predictions [65]. These advancements highlight the dual potential of DFT for both optimizing co-reactants and guiding the rational design of novel luminophore frameworks.

In summary, our work serves as a paradigm for expanding the applications of SACs in ruthenium-ECL systems, offering a promising strategy for designing highly active and selective ECL promoters. We lay a solid foundation for the clinical implementation and future development of advanced IVD techniques.

## Materials and Methods

### Materials

Cobalt (II) chloride hexahydrate (CoCl_2_·6H_2_O, 98%), phenol (0.1 M), hydrochloric acid (HCl) melamine (C_3_H_6_N_6_), graphene powder, and isopropanol were all purchased from Sigma-Aldrich. All experiments were conducted using deionized water (DW). Ru(bpy)_3_^2+^ was sourced from Suna Tech Inc. Isopropanol was sourced from Sinopharm Chemical Reagent Co. Ltd., while N-hydroxysuccinimide (NHS, GR) and 1-ethyl-3-(3-dimethylaminopropyl) carbodiimide hydrochloride (EDC, GR) were purchased from Shanghai Medpep Co. Ltd. (Shanghai, China). Bovine serum albumin (BSA; 96% to 99%, GR) was supplied by Biss Inc. (Beijing, China), and CEA along with its antibodies was obtained from Beyotime Biotechnology Co. Ltd. (Shanghai, China). Human serum samples were provided by Chongqing Medical University. ECL detection was performed in phosphate-buffered saline (PBS, 0.1 M, pH 6) prepared with KH₂PO₄, Na₂HPO₄, and KCl (0.1 M) in appropriate ratios. Unless otherwise stated, all reagents were of analytical grade and used without further purification. Aqueous solutions were freshly prepared and diluted with ultrapure water (≥18 MΩ; Milli-Q, Millipore) as required.

### Catalysts synthesis

First, commercial graphene powder was dissolved in 30 ml of methanol solution with vigorous stirring. Then, 3 ml of cobalt solution in water (CoCl_2_·6H_2_O, 2 mol l^−1^) was slowly added, and the mixture was stirred in the dark for at least 1 h until the Co precursor and graphene were dispersed evenly. Subsequently, the mixed solution was vigorously stirred at 60 °C until dry to obtain a homogeneous mixture of the Co precursor and graphene. Afterwards, the 2 composites were heated at 900 °C for 24 h in a N_2_ atmosphere with or without melamine. The as-prepared CoC₄@graphene and CoN₄@graphene were treated in 1 M hydrochloric acid for 36 and 24 h, respectively, to eliminate Co nanoparticle by-products. The as-obtained precipitates were centrifuged several times with DW. After these steps, CoC_4_@graphene and CoN_4_@graphene were successfully prepared.

### Apparatus and characterizations

ECL measurements were carried out on an MPI-E multifunctional ECL analyzer (Xi’an Remex Analytical Instrument Ltd. Co., China) using a 3-electrode configuration, consisting of a modified glassy carbon working electrode (φ = 3 mm), an Ag/AgCl (saturated KCl) reference electrode, and a platinum wire counter electrode. The photomultiplier tube (PMT) was set to 600 V, with a scan voltage ranging from 1.5 to −2 V at a scan rate of 100 mV s^−1^.

For material characterization, XPS was performed with a VG Multilab 2000X system (Thermal Electron, USA), Fourier transform infrared (FT-IR) spectra were obtained using an ALPHA spectrometer (Bruker), and TEM images were captured on a JEM-2010 microscope (JEOL, Japan). XAFS data processing, including background correction and FT fitting, was conducted using Athena and Artemis (version 0.9.26) [66,67], with k^2^-weighting and optimized parameters: Co-foil (k-range: 3 to 11.5 Å^−1^, R-range: 1 to 3 Å) and Sample (k-range: 3 to 12 Å^−1^, R-range: 1 to 2 Å). WT analysis was carried out via the Hama Fortran code, utilizing χ(k) data exported from Athena and a Morlet mother wavelet function (κ = 10, σ = 1) with an R-range of 0 to 4 Å and k-range of 0 to 16 Å^−1^. Raman spectra were recorded using a Renishaw InVia spectrometer with a model 100 RamaScope optical fiber system.

### Electrochemical measurements

Electrochemical analyses, including CV and EIS, were conducted using an Ivium electrochemical workstation (Netherlands). EIS measurements were acquired by applying a 5-mV perturbation over a frequency range of 0.01 to 10^6^ Hz, in a 5 mM K₃[Fe(CN)₆]/K₄[Fe(CN)₆] solution containing 0.1 M KCl. The ECSA of the catalyst was estimated based on the electrochemical double layer capacitance (*C*_dl_), derived from the CVs measured within the non-Faradic potential range at the different sweeping rates (v). *C*_dl_ was obtained by plotting half the difference between the anode and cathode current densities, whose slope corresponds to *C*_dl_. The ECSA of the catalyst was estimated according to the equation:

$$\text{ECSA}=\frac{C_{\mathrm{dl}}}{C_{s}}$$ (18)

where *C*_s_ is the roughness factor with a value of 40.0 μF cm^−2^.

The ECSA-normalized LSVs were obtained through the equation:

$$J_{\text{normalized}}=\frac{J}{\text{ECSA}}$$ (19)

where J and $J_{\text{normalized}}$ are the measured and ECSA-normalized current densities, respectively.

For ORR, all tests were carried out in a 3-electrode electrochemical system using CHI 760E, with Ag/AgCl (saturated KCl solution) and platinum wire as the reference electrode and counter electrode, respectively. To prepare the working electrode, 4 mg of the catalyst was ultrasonically dispersed into the 1-ml mixed solution of distilled water (700 μl), ethanol (250 μl), and Nafion117 solution (5%, 50 μl) to form a homogeneous ink. Then, 5 μl of the ink was dropped onto the GCE with a disk diameter of 5 mm, placed at room temperature until dry. The corresponding catalyst loading is 0.1 mg cm^−2^. The CV test was carried out in O_2_-saturated 0.1 M KOH and the data were recorded at a scan rate of 50 mV s^−1^ under static conditions after the system became stable. The RDE and RRDE tests were conducted in O_2_-saturated 0.1 M KOH with a scan rate of 10 mV s^−1^.

The electron transfer number (*n*) is calculated by the following equation:

$$x=\frac{4\left|I_{d}\right|}{\left|I_{d}\right|+I_{r}/N}$$ (20)

$$H_{2}O_{2}\%=\frac{200\left(I_{r}/N\right)}{\left|I_{d}\right|+I_{r}/N}$$ (21)

where $I_{d}$ represents the disk current, $I_{r}$ stands for the ring current, and *N* is the current collection efficiency of the Pt ring, which was 0.37.

The K–L plot ($J^{-1}\;\mathrm{vs}.\omega^{-1/2}$) was calculated at different potentials. The working electrode was scanned cathodically at a rate of 10 mV s^−1^ with the rotation speed from 225 to 3,600 rpm.

K–L equation:

$$\frac{1}{J}=\frac{1}{J_{L}}+\frac{1}{J_{K}}=\frac{1}{B\omega^{1/2}}+\frac{1}{J_{K}}$$ (22)

$$B=0.2\mathrm{nF}C_{0}D_{0}^{2/3}v^{-1/6};J_{k}=\mathrm{nFk}C_{0}$$ (23)

where J is the measured current density, $J_{K}\;$is the kinetic current density and $J_{L}$ is the limiting current density, ω is the angular velocity, n is transferred electron number, F (96,485 C mol^−1^) is the Faraday constant, $D_{0}$ is the diffusion coefficient of O_2_ in 0.1 M KOH (1.9×10^-5^ cm s^−1^), $C_{0}$ is the bulk concentration of O_2_ (1.2×10 ^-6^ cm s^−1^), v is the kinetic viscosity of the electrolyte (0.01 cm s^−1^), and k is the electron transfer rate constant. The constant 0.2 is adopted when the rotation speed is shown in rpm.

The following formula was used to give a rough estimation of the TOF:

$$\mathrm{TOF}=\frac{I}{\mathrm{nFN}}$$ (24)

where I refers to the LSV current measured at 0.8 V vs. RHE. *n* is the electron transfer number during the ORR process (3 for CoC_4_@graphene and 4 for CoN_4_@graphene). *F* is Faraday constant (96,485 C mol^−1^) and *N* represents moles of active site (mol).

MA is calculated based on the following equation:

$$\mathrm{MA}=\frac{I}{m}$$ (25)

where *I* is the LSV current measured at 0.8 V vs. RHE, and *m* refers to the actual mass of catalyst loaded on the electrode, which was calculated by multiplying the mass of the deposited catalyst by the mass fraction of Co determined via ICP-MS.

### Computational details

This study utilized spin-polarized DFT calculations with the Vienna Ab Initio Simulation package, employing plane-wave basis sets and projector-augmented wave pseudopotentials. The Perdew–Burke–Ernzerhof exchange correlation functional within the framework of Generalized Gradient Approximation was applied. A plane-wave cutoff of 400 eV was set, and the Brillouin zone was sampled using a 2 × 2 × 1 Monkhorst-Pack k-point grid for structural optimization. Convergence of electron density for ground states was achieved with a total energy threshold of 10^−4^ eV, and structure optimization continued until the maximum force on any ion was below 0.05 eV Å^−1^. A vacuum space of approximately 20 Å along the *z*-direction was implemented to prevent interactions between the periodic substrate and repeatable images. For the N-doped system, a 4 × 4 graphene super cell was utilized, with the Co atom embedded at the double vacancy (CoC*_x_*N_4−*x*_, *x* = 0 to 4).

Four reaction steps in 4e^−^ORR pathway are listed as follows:

O2+∗+H++e−→O2H∗ (26)

O2H∗+H++e−→O∗+H2O (27)

O∗+H++e−→OH∗ (28)

OH∗+H++e−→∗+H2O (29)

Four reaction steps in HPRR pathway are listed as follows:

H2O2+∗→H2O2∗ (30)

H2O2∗→2OH∗ (31)

OH∗+H++e−→H2O+∗ (32)

OH∗+H++e−→H2O+∗ (33)

### Construction of immunosensor

CoN_4_@graphene was mixed with the Ab_2_ and incubated for 12 h to obtain the label. Then, 5 μl of 2 mg ml^−1^ CoC_4_@graphene was dropped onto the polished electrode surface, followed by the addition of 5 μl of EDC/NHS, with incubation at 37 °C for 30 min. After washing, primary antibodies (Ab_1_) were added and incubated at 37 °C for 1 h. Following another washing step, we use BSA to block the sensor, and antigen and the prepared secondary antibody were sequentially added. After these steps, the immunosensor was successfully constructed and can be subsequently employed for the detection of biomarkers.

## Data Availability

Data supporting the findings of this study are available in the main text or the Supplementary Materials. Additional data related to this paper may be requested from the authors.
