# Supplementary material for: Precision-Engineered Co-N4−x-Cx Single Atoms Enhance Potential-Resolved Ru(bpy)32+ Electrochemiluminescence via Reactive Oxygen Species
Source: Research (Wash D C). 2025 Aug 14;8:0842. doi: 10.34133/research.0842 (PMC12351920; doi:10.34133/research.0842)
Supplement: Supplementary 1 — Figs. S1 to S16 Tables S1 to S6 [file research.0842.f1.docx]

Supplementary Information for

Precision-Engineered Co-N_4-x_-C_x_ Single Atoms Enhance Potential-Resolved Ru(bpy)_3_^2+^ Electrochemiluminescence via Reactive Oxygen Species

Ziqi Kang^1†^, Shu Zhu^1†^, Shijun Wang^1†^, Zhizhi Xiang^1^, Zixin Deng^1^, Xuehao Tong^1^, Zixu Wang^1^, Yanghan Sun^1^, Xiancheng Liu^3,4^, Guangchao Zang^1,4,5*^, Chenzhong Li^6^*, Guixue Wang^3,4*^, Yuchan Zhang^1,2*^

^1^ Biomedical Innovation and Entrepreneurship Practice Base, Lab Teaching & Management Center, Chongqing Medical University, Chongqing 400016, China.

^2^ College of Biomedical Engineering, Chongqing Medical University, Chongqing 400016, China.

^3^ Key Laboratory for Biorheological Science and Technology of Ministry of Education, National Local Joint Engineering Lab for Vascular Implants, College of Bioengineering, Chongqing University, Chongqing 400044, China.

^4^ JinFeng Laboratory, Chongqing 401329, China.

^5^ Western Institute of Digital-Intelligent Medicine, Chongqing 401329, China.

6 Bioelectronics and Biosensors Center, School of Medicine, Chinese University of Hong Kong, Shenzhen, 2001 Longxiang Avenue, Longgang District, Shenzhen, 518172, China.

^*^ Correspondence: [zangguangchao@cqmu.edu.cn](mailto:zangguangchao@cqmu.edu.cn) (G.Z.); [lichenzhong@cuhk.edu.cn](mailto:lichenzhong@cuhk.edu.cn)(C.L.) ; [wanggx@cqu.edu.cn](mailto:wanggx@cqu.edu.cn) (G.W.); [zhangyc@cqmu.edu.cn](mailto:zhangyc@cqmu.edu.cn) (Y.Z.).

† These authors contributed equally to this work.

**1. Supplementary figures**


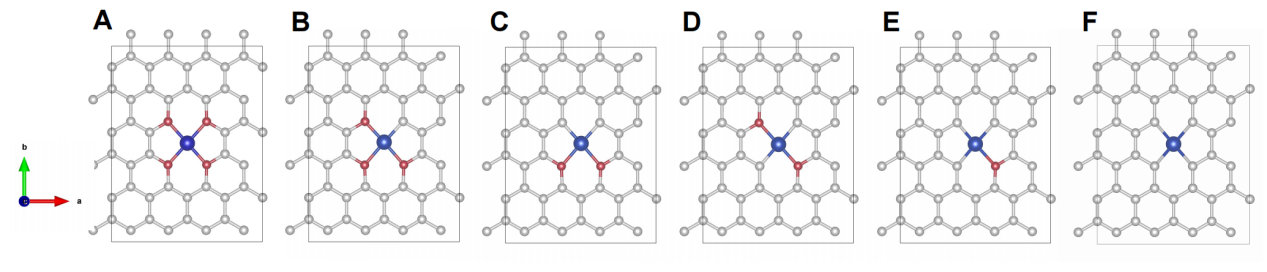


**Fig. S1.** Co single-atom catalysts with different C/N ratios, including (A) CoN_4_, (B) CoC_1_N_3_, (C) CoC_2_N_2_-1, (D) CoC_2_N_2_-2, (E) CoC_3_N_1_, and (F) CoC_4_.


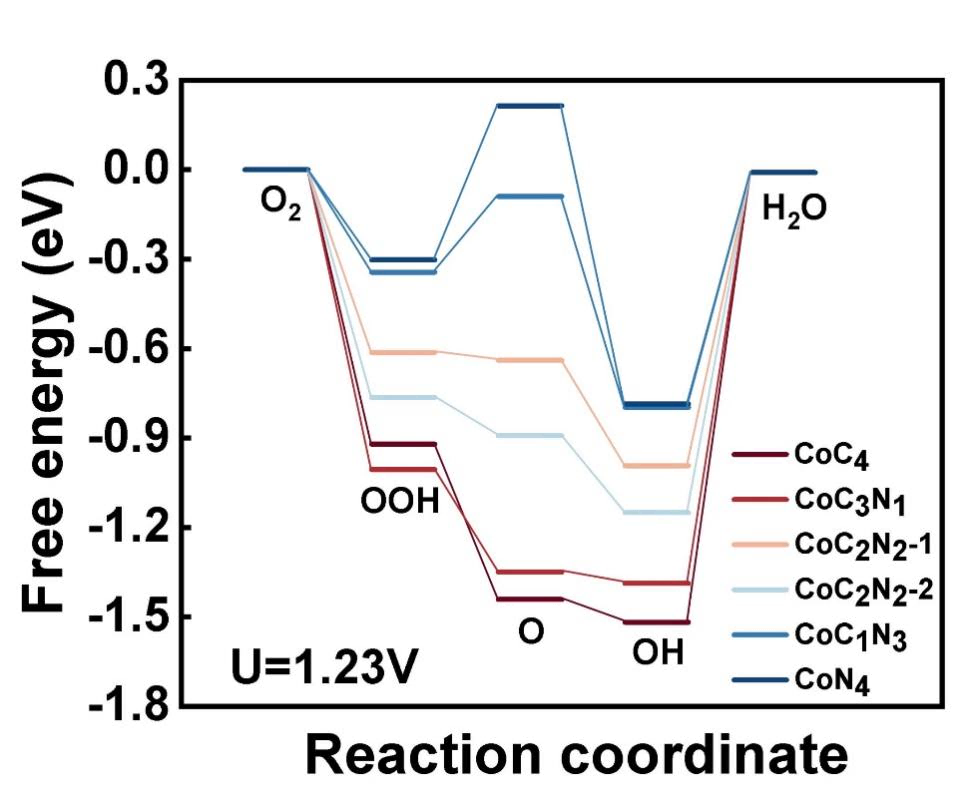


**Fig. S2.** Calculated free energy diagrams for ORR at U=1.23V of the Co atom of CoC_x_N_4-x_.


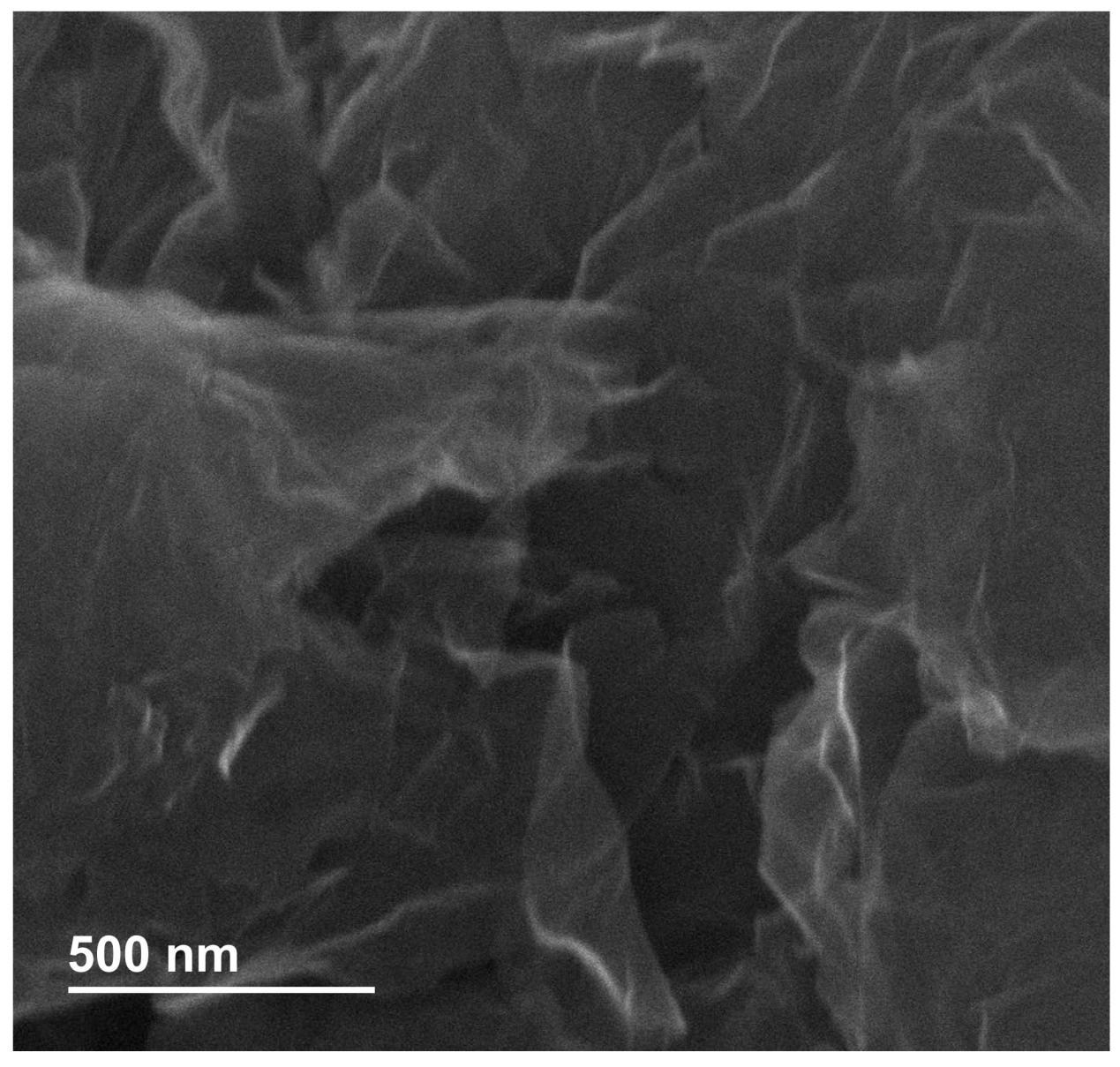


**Fig. S3.** SEM images of graphene.


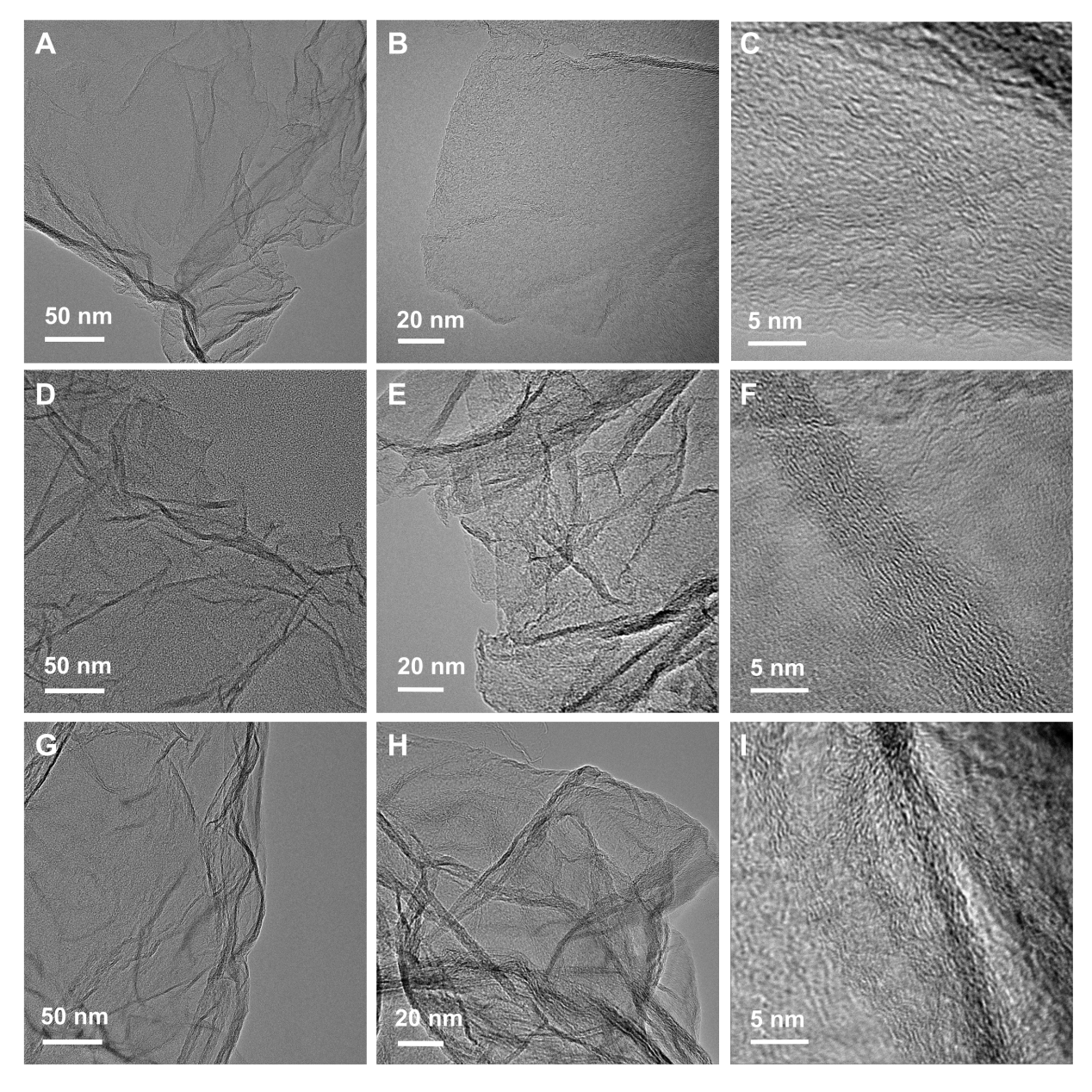


**Fig. S4.** TEM images of (A-C) CoC_4_@graphene, (D-F) CoN_4_@graphene and (G-I) graphene at different scales.


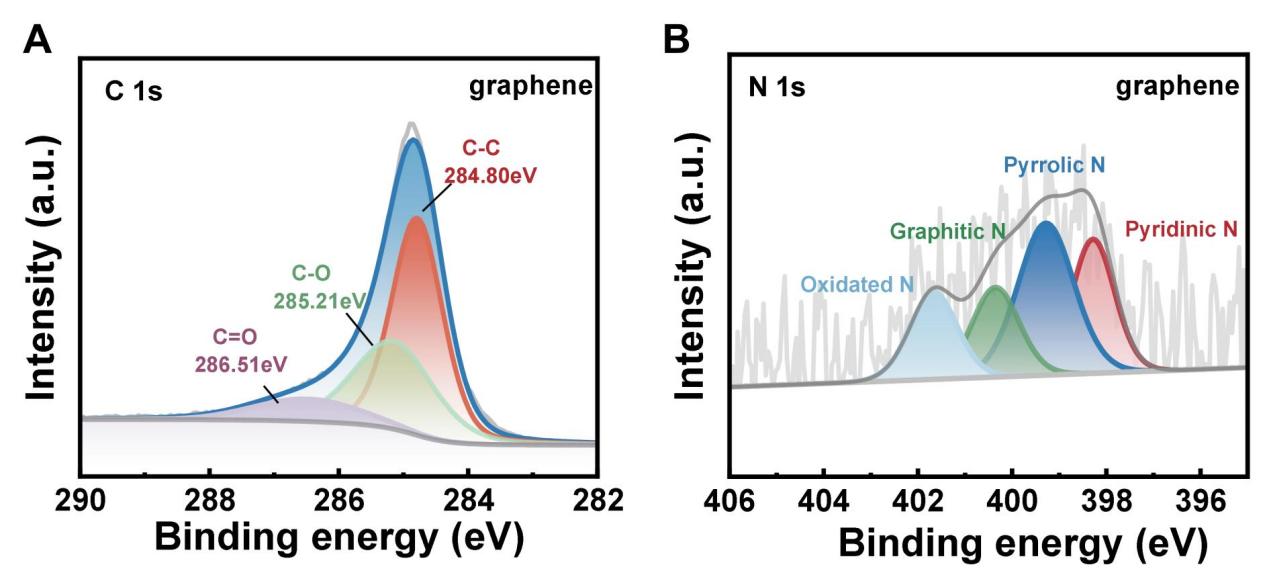


Fig. S5. XPS spectrum of (A) C 1s deconvolution and (B) N 1s deconvolution for graphene.


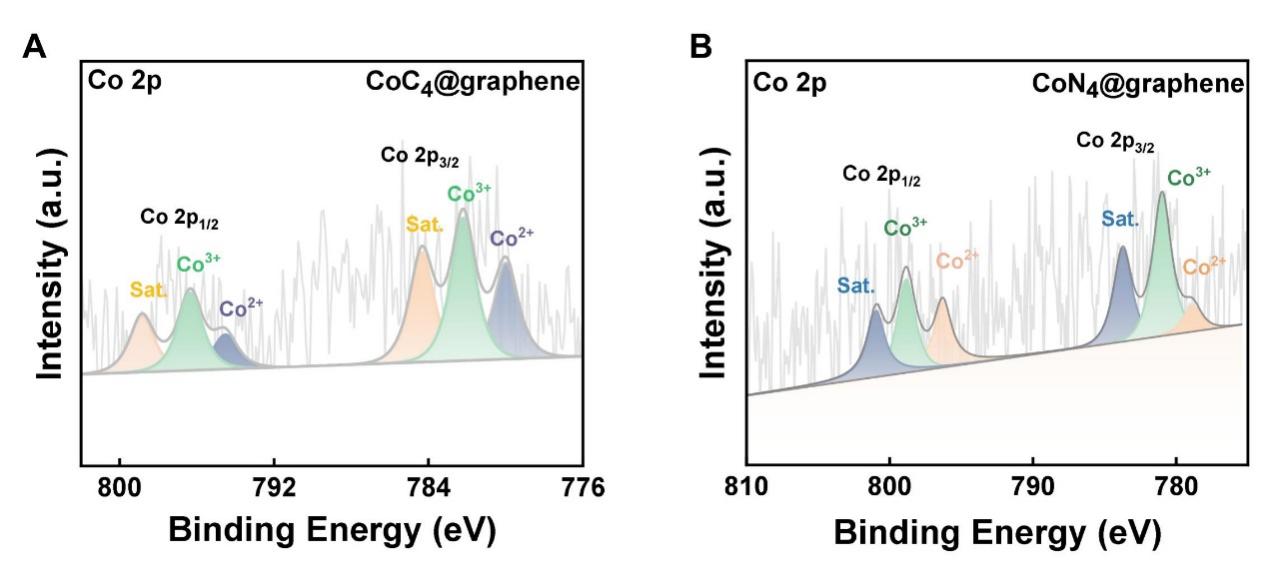


Fig. S6. XPS spectrum of Co 2p deconvolution for (A) CoC_4_@graphene and (B) CoN_4_@graphene.


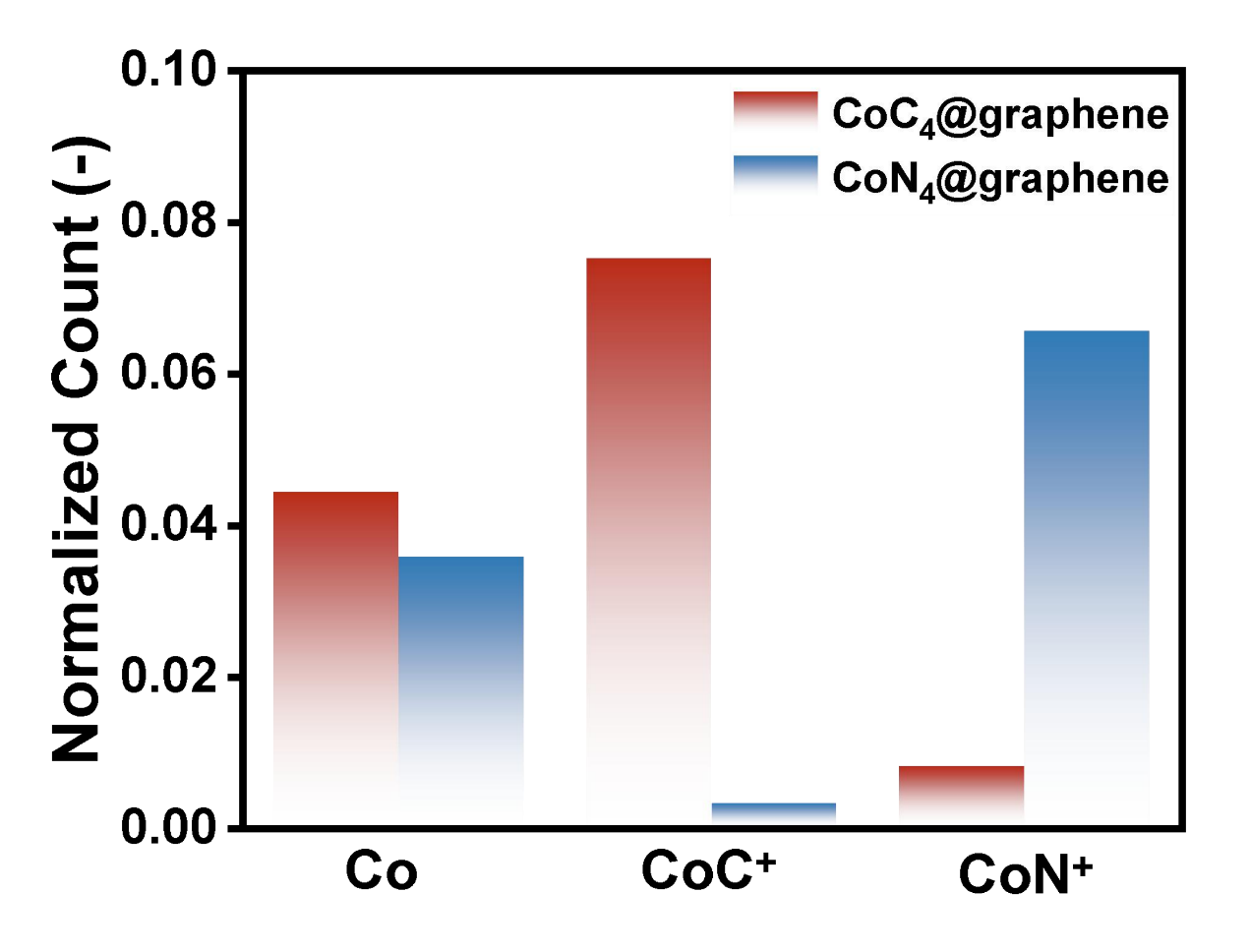


Fig. S7. ToF-SIMS positive spectrum results of CoC4@graphene and CoN4@graphene. Fragment counts were normalized to the total ion count.


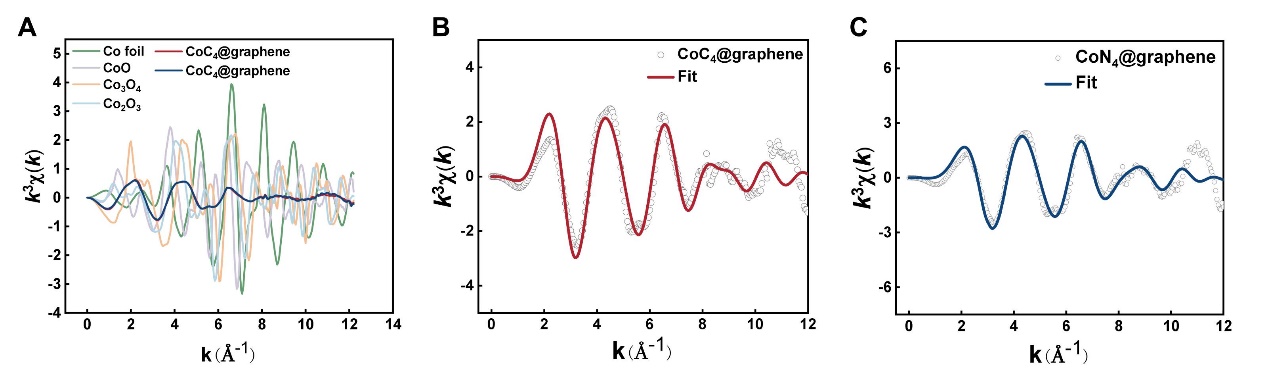


Fig. S8. (A) k space EXAFS curves. Corresponding Co K-edge EXAFS fitting curves of (B) CoC_4_@graphene and (C) CoN_4_@graphene.


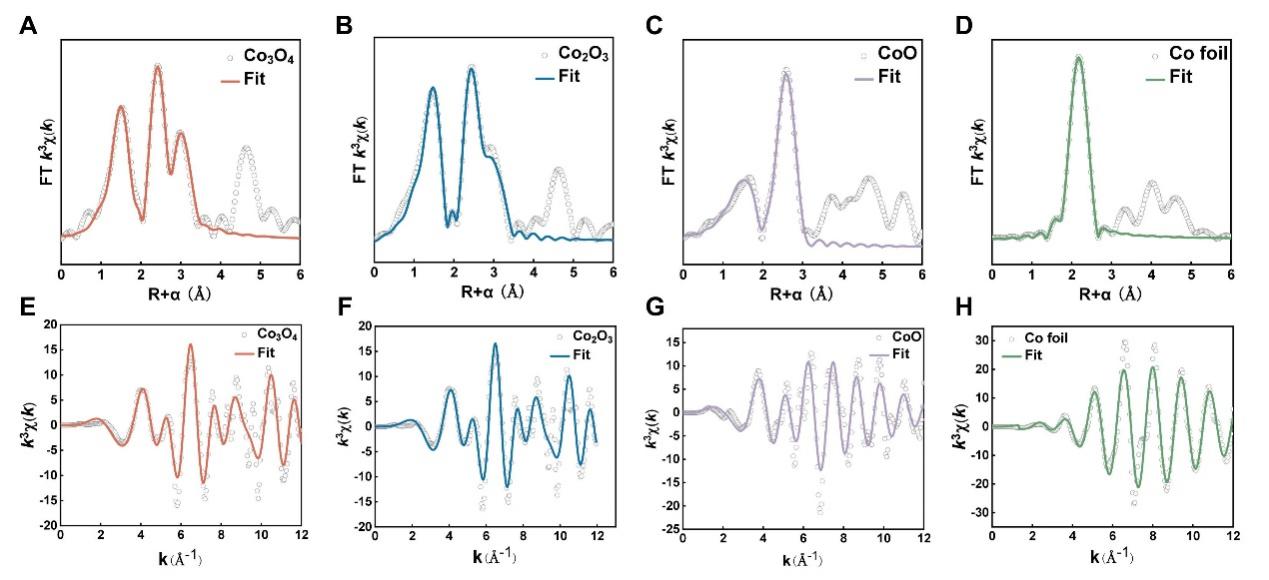


Fig. S9. Corresponding Co K-edge EXAFS fitting curves of (A) Co_3_O_4_, (B) Co_2_O_3_, (C) CoO and (D) Co foil in R space. Corresponding Co K-edge EXAFS fitting curves of (E) Co_3_O_4_, (F) Co_2_O_3_, (G) CoO and (H) Co foil in k space.


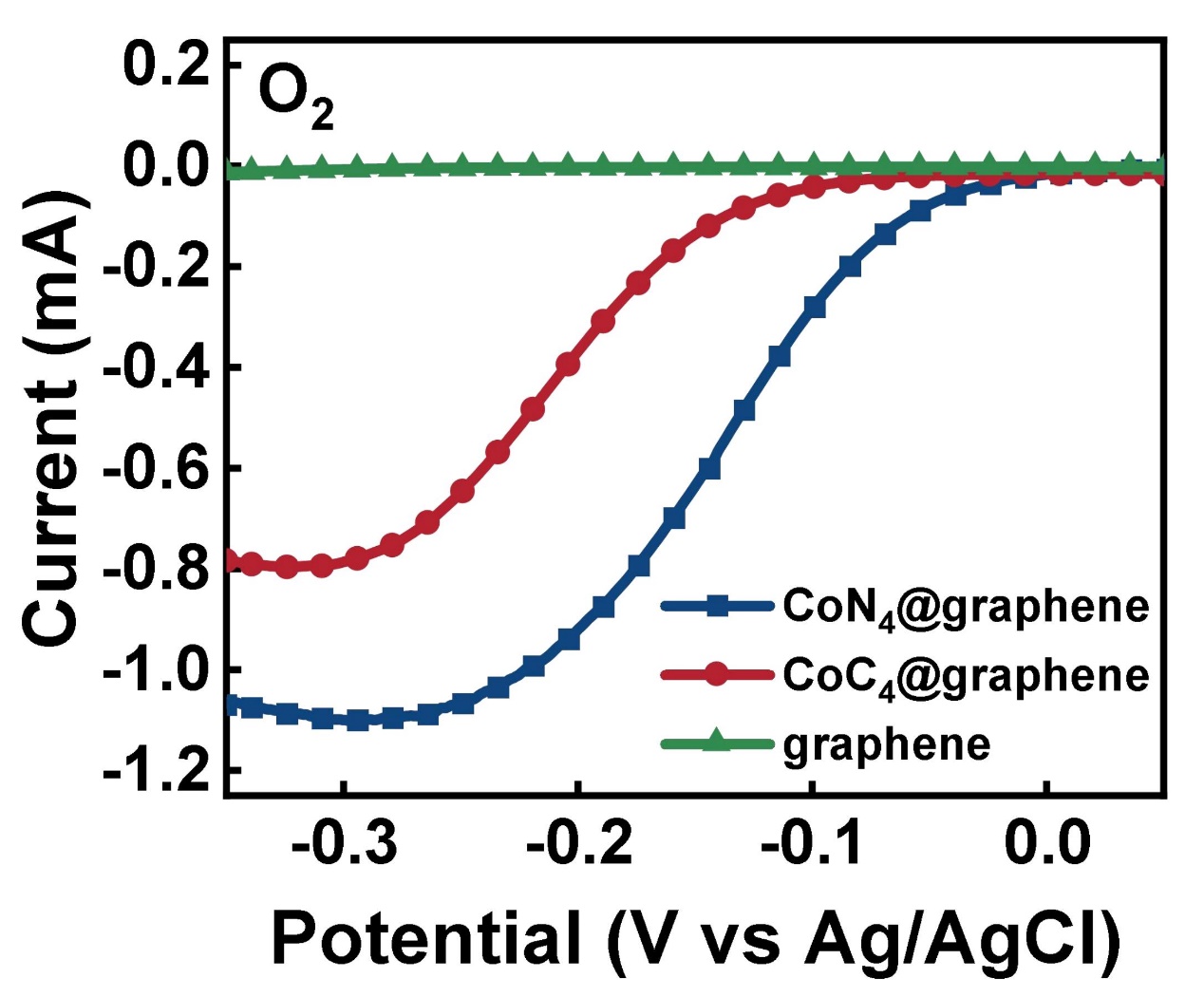


Fig. S10. LSV current curves of catalysts at the 1600 rpm in 0.1M KOH in O_2_-saturated atmosphere.


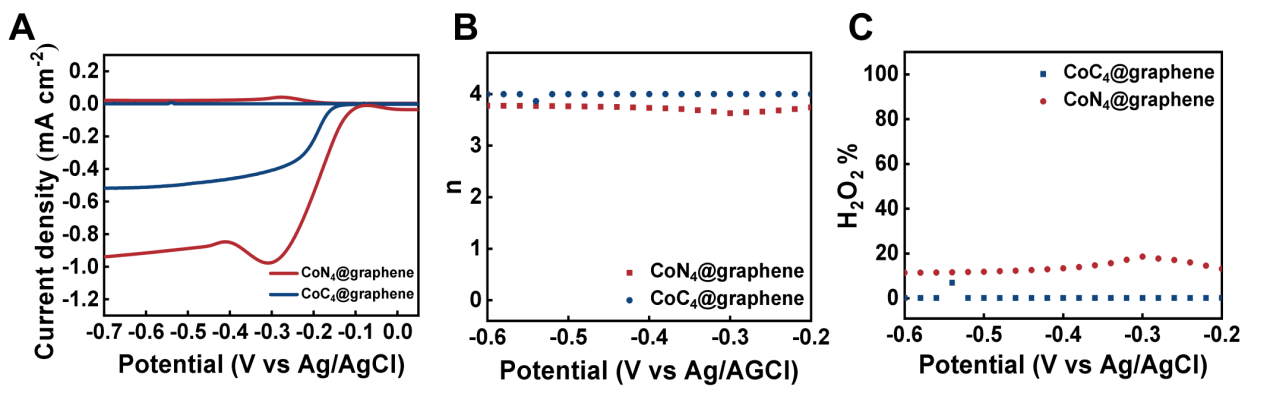


Fig. S11. (A) The ring current and disk current of RRDE at 1,600 rpm in O_2_-saturated 0.1 M KOH solution. (B) Average electron transfer number *(n)* and (C) H_2_O_2_ selectivity during the ORR of CoN_4_@graphene (red line) and CoC_4_@graphene (blue line).

The RRDE quantifies the number of transferred electrons and the H_2_O_2_ generation rate by analyzing the percentage contributions of the four-electron process (reflected by the disk current, I_d_) and the two-electron process (reflected by the ring current, I_r_) to the overall reaction process. This approach provides an accurate evaluation of CoN_4_@graphene but fails to precisely reflect the number of transferred electrons involved in the ORR for CoC_4_@graphene due to its predominant 3e^-^ORR and rare 2e^-^ORR (theoretical calculations suggest that •OOH to H_2_O_2_ is an energy-consuming process with a ΔG_5_ greater than ΔG_4_ for the 4e^-^ORR).


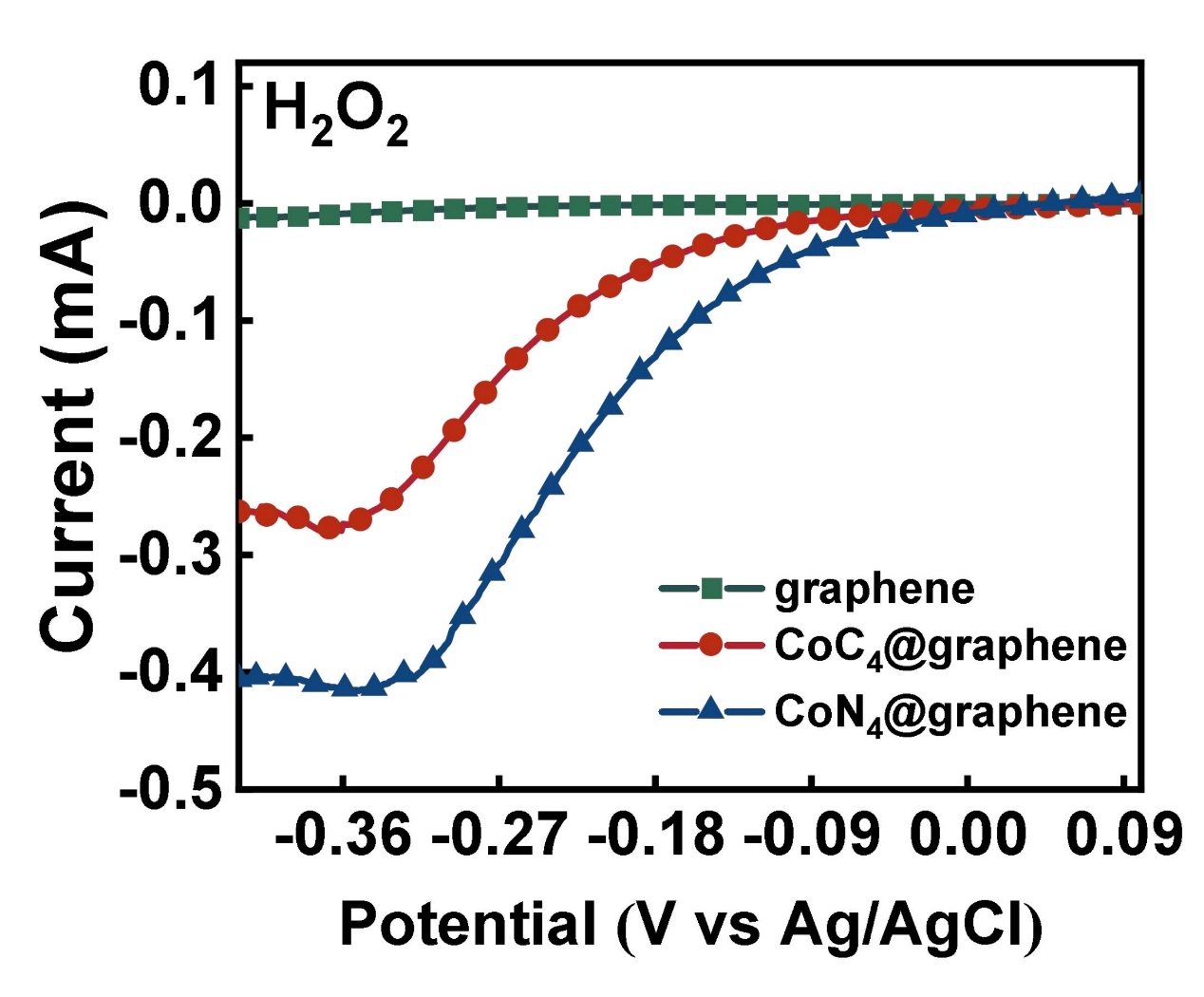


Fig. S12. LSV current curves of catalysts at the 1600 rpm in 0.1M KOH and 5mM H_2_O_2_ in N_2_-saturated atmosphere.


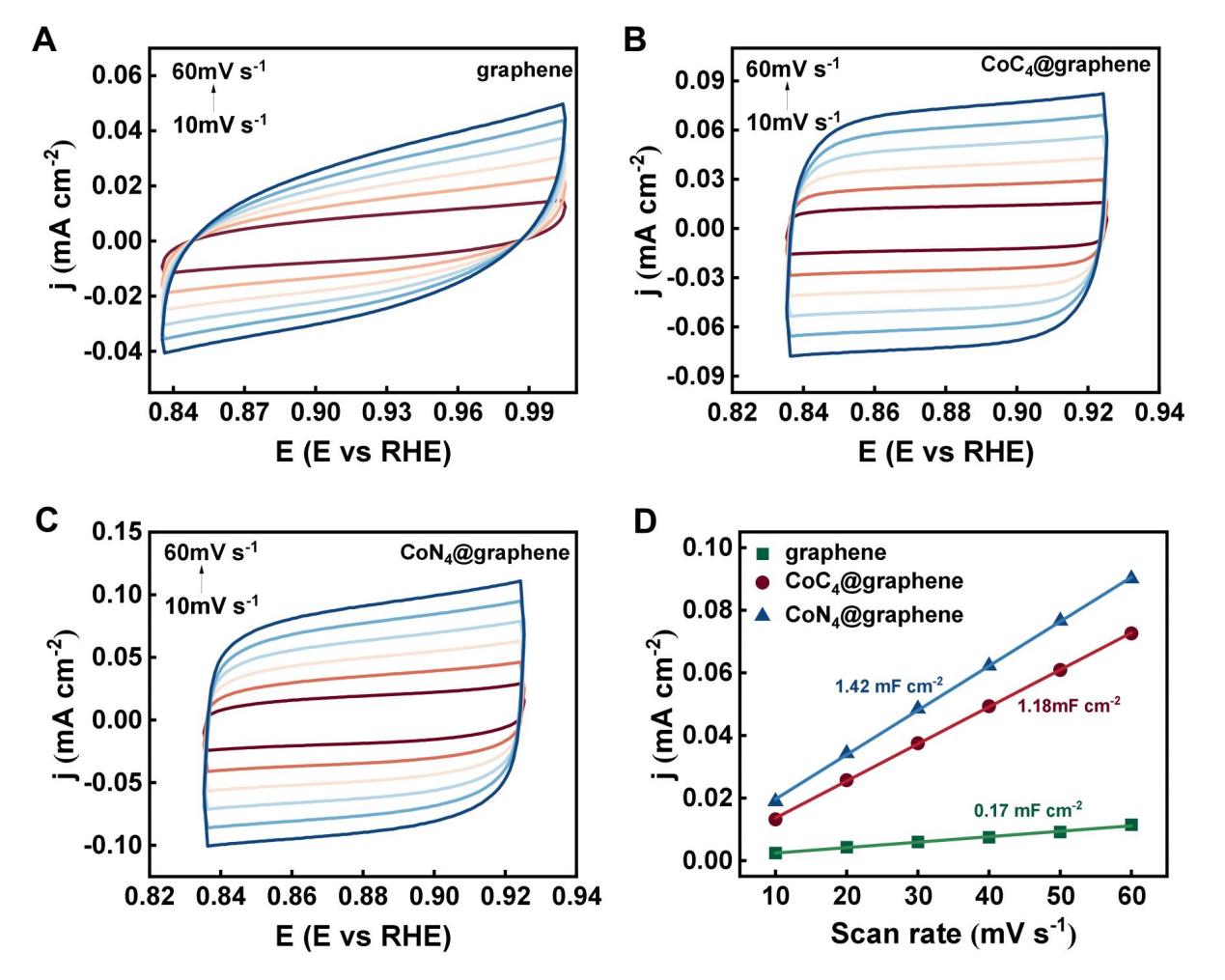


Fig. S13. CVs of the catalysts at different scan rates from 10 to 60 mV s^-1^ of (A) graphene, (B) CoC_4_@graphene and (C) CoN_4_@graphene. (D) C_dl_ plot of the catalysts.


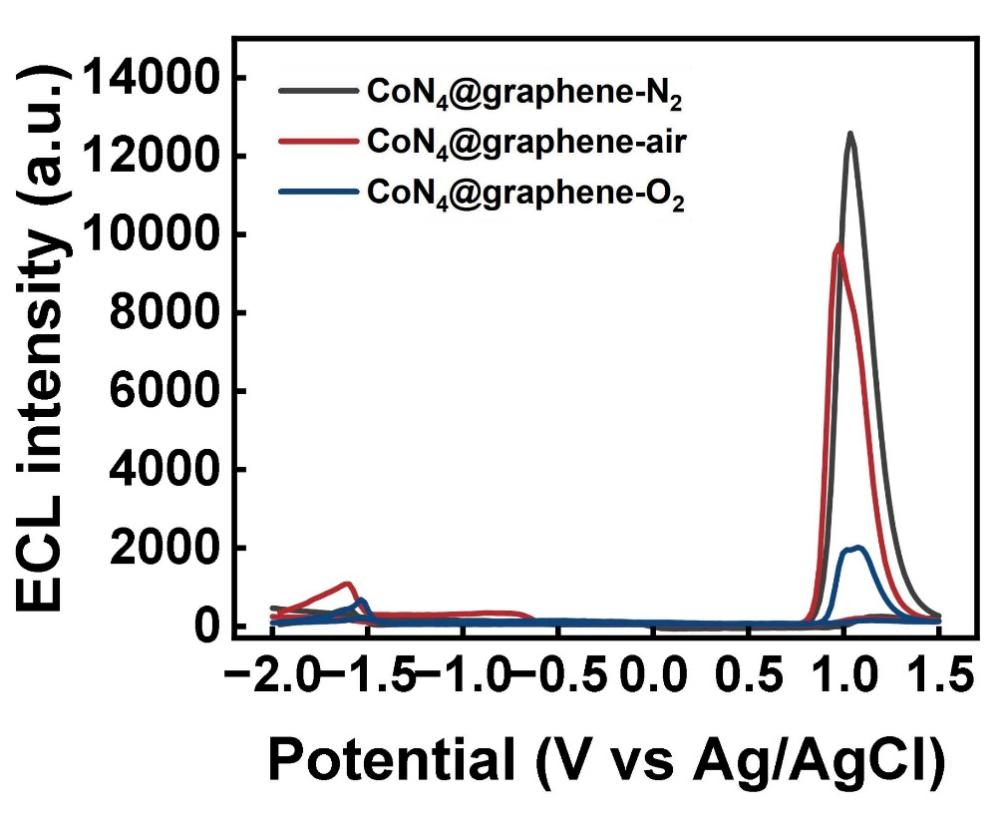


**Fig. S14.** ECL lines of CoN_4_@graphene in N_2_ (dark gray line), air (red line) and O_2_ (dark blue line) in 1 mM Ru(bpy)_3_^2+^ solution (−2 to 1.5 V versus Ag wire, scan rate of 0.1 V s^-1^).


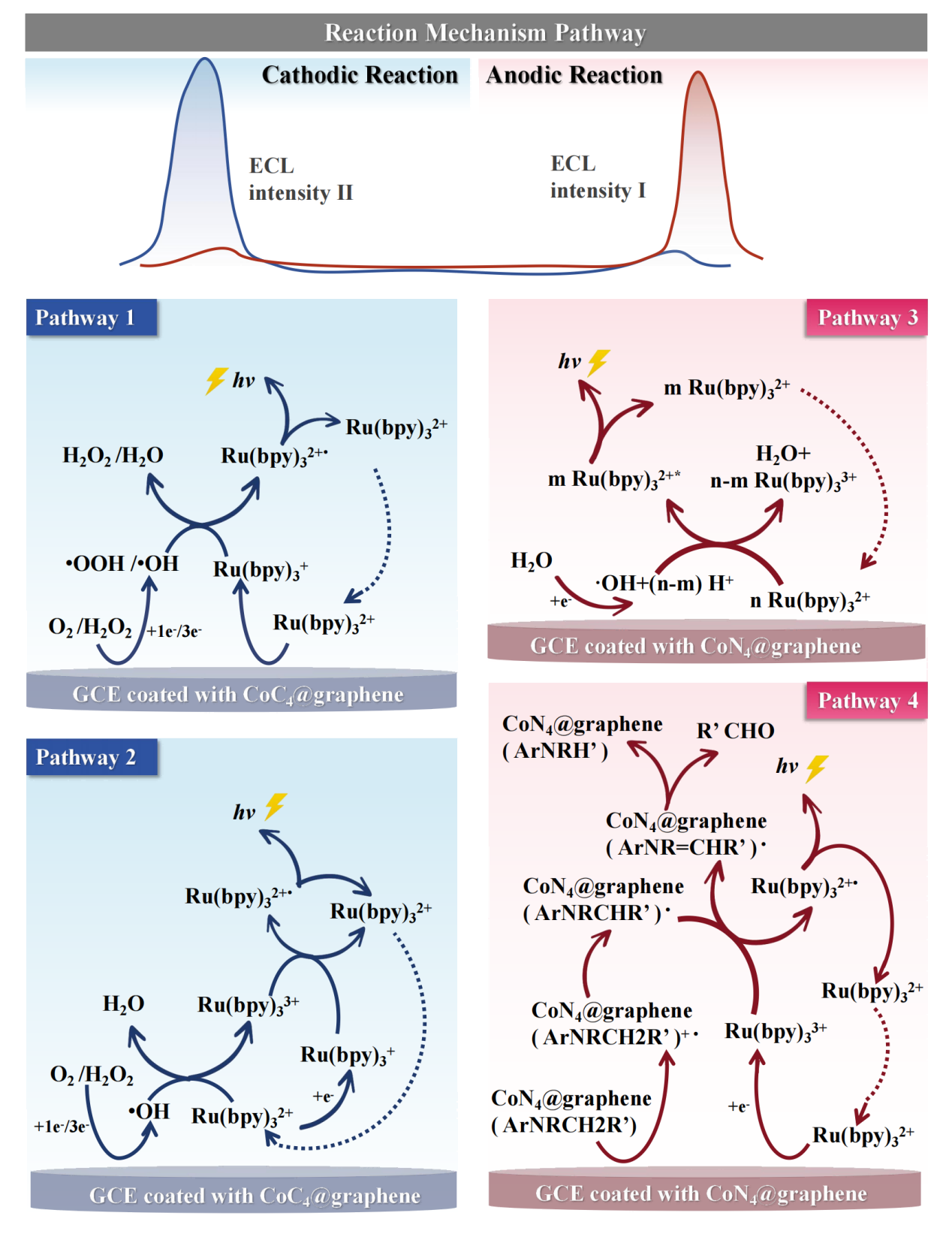


**Fig. S15.** The schematic illustration for reaction mechanism pathways of cathodic ECL generation on CoC_4_@graphene/GCE (blue) and anodic ECL generation on CoN_4_@graphene/GCE (red) in Ru(bpy)_3_^2+^ with PBS at pH=6.


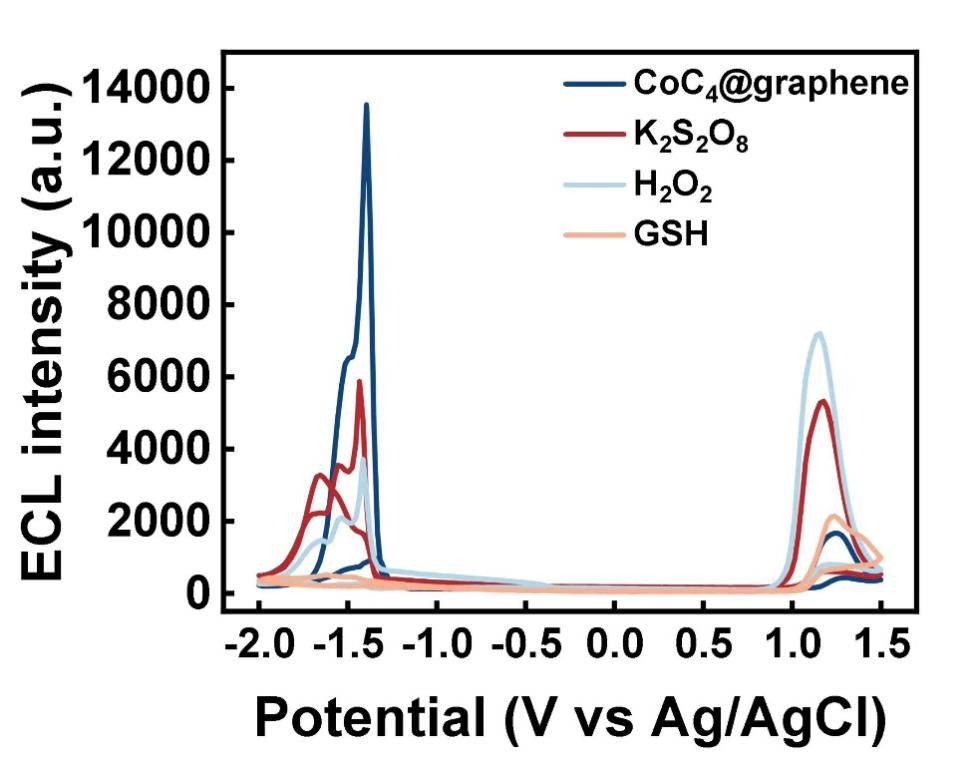


**Fig. S16.** The comparison of the cathodic ECL performance of Ru(bpy)_3_^2+^ with CoC_4_@graphene (dark blue line) and traditional Ru(bpy)_3_^2+^’s cathodic co-reactant GSH (orange line), K_2_S_2_O_2_(red line), H_2_O_2_ (light blue line).

**2. Supplementary Table**

**Table S1**. The Gibbs free energy for ORR at U=0 V of CoC_x_N_4-x_.

|  | CoN_4_ | CoC_1_N_3_ | CoC_2_N_2_-1 | CoC_2_N_2_-2 | CoC_3_N_1_ | CoC_4_ |
| --- | --- | --- | --- | --- | --- | --- |
| Δ*G*_1_ | -1.5325 | -1.5738 | -1.9934 | -1.8428 | -2.2354 | -2.1507 |
| Δ*G*_2_ | -0.713 | -0.9761 | -1.3586 | -1.2568 | -1.5743 | -1.7511 |
| Δ*G*_3_ | -2.2299 | -1.9369 | -1.4883 | -1.5832 | -1.2678 | -1.3062 |
| Δ*G*_4_ | -0.4546 | -0.4432 | -0.0897 | -0.2472 | 0.1475 | 0.278 |
| Δ*G*_5_ | -0.3057 | -0.2261 | 0.1947 | 0.0446 | 0.4385 | 0.366 |

**Table S2**. The Gibbs free energy for ORR at U=1.23 V of CoC_x_N_4-x_.

|  | CoN_4_ | CoC_1_N_3_ | CoC_2_N_2_-1 | CoC_2_N_2_-2 | CoC_3_N_1_ | CoC_4_ |
| --- | --- | --- | --- | --- | --- | --- |
| Δ*G*_1_ | -0.3025 | -0.3438 | -0.7634 | -0.6128 | -1.0054 | -0.9207 |
| Δ*G*_2_ | 0.517 | 0.2539 | -0.1286 | -0.0268 | -0.3443 | -0.5211 |
| Δ*G*_3_ | -0.9999 | -0.7069 | -0.2583 | -0.3532 | -0.0378 | -0.0762 |
| Δ*G*_4_ | 0.7754 | 0.7868 | 1.1403 | 0.9828 | 1.3775 | 1.508 |
| Δ*G*_5_ | 0.9243 | 1.0039 | 1.4247 | 1.2746 | 1.6685 | 1.596 |

**Table S3.** The Gibbs free energy for HPRR at U=1.23 V of CoC_x_N_4-x_.

|  | CoN_4_ | CoC_1_N_3_ | CoC_2_N_2_-1 | CoC_2_N_2_-2 | CoC_3_N_1_ | CoC_4_ |
| --- | --- | --- | --- | --- | --- | --- |
| Δ*G*_6’_ | -0.4382 | -0.3999 | -0.3987 | -0.3982 | -0.3969 | -0.3847 |
| Δ*G*_7_ | -0.3542 | -0.7868 | -1.7025 | -1.5756 | -2.0726 | -2.4797 |
| Δ*G*_8_ | 0.7754 | 0.7868 | 1.1403 | 0.9828 | 1.3775 | \| 1.508 \| \| --- \| |

**Table S4.** Elemental contents determined by ICP-MS for CoC_4_@graphene and CoN_4_@graphene.

|  | Sample mass  (g) | constant volume  (mL) | Dilution factor | element | Instrument reading  (mg L^-1^) | Converted content  (mg kg^-1^) | Unit Mass fraction  (%) |
| --- | --- | --- | --- | --- | --- | --- | --- |
| CoC_4_@graphene | 0.0101 | 25 | 1 | Co | 0.6739 | 1668.1 | 0.1668% |
| CoN_4_@graphene | 0.0039 | 25 | 1 | Co | 0.4247 | 2722.5 | 0.2723% |

**Table S5.** The EXAFS fitting parameters of Co R-space for CoC_4_@graphene, CoN_4_@graphene, Co foil, CoO, Co_2_O_3_, Co_3_O_4_.

|  | shell | CN*^a^* | R*^b^*(Å) | σ^2^*^c^*(Å^2^) | ΔE_0_*^d^*(eV) | R factor |
| --- | --- | --- | --- | --- | --- | --- |
| CoC_4_@graphene | Co-C | 3.5±0.6 | 1.99±0.01 | 0.0141 | -0.1±2.4 | 0.0120 |
|  | Co-C-C | 1.0 | 2.86±0.01 | 0.0030 |  |  |
| CoN_4_@graphene | Co-N | 4.5±0.7 | 1.94±0.01 | 0.0131 | 0.9±1.9 | 0.0063 |
|  | Co-N-C | 0.8 | 2.86±0.01 | 0.0030 |  |  |
| Co-foil | Co-Co | 12.0 | 2.49±0.01 | 0.0063 | 6.9±0.3 | 0.0007 |
| CoO | Co-O | 2.9±0.7 | 2.09±0.01 | 0.0177 | -2.3±0.8 | 0.0094 |
|  | Co-Co | 12.3±0.3 | 3.01±0.01 | 0.0095 |  |  |
| Co_2_O_3_ | Co-O | 4.6±0.5 | 1.92±0.08 | 0.0025 | -4.8±1.0 | 0.0097 |
|  | Co-Co1 | 1.4±0.2 | 2.88±0.01 | 0.0075 |  |  |
|  | Co-Co2 | 2.0±0.1 | 3.38±0.01 | 0.0032 |  |  |
| Co_3_O_4_ | Co-O | 4.2±0.8 | 1.93±0.07 | 0.0020 | -3.2±2.5 | 0.0131 |
|  | Co-Co1 | 3.4±0.7 | 2.85±0.03 | 0.0012 |  |  |
|  | Co-Co2 | 10.6±1.5 | 3.17±0.04 | 0.0085 |  |  |

*^a^CN*: coordination numbers; *^b^R*: bond distance; *^c^σ*^2^: Debye-Waller factors; *^d^* Δ*E*_0_: the inner potential correction. R factor: goodness of fit. Error bounds that characterize the structural parameters obtained by EXAFS spectroscopy were estimated as CN±20%; R ± 1%; σ^2^ ± 20%.

**Table S6.** Immunoassay of CEA in real samples using a spiked method in our Co SACs-based ratio immunosensor.

| Sample | CEA Added  (g mL^-1^) | CEA detected  (g mL^-1^) | RSD (%) |
| --- | --- | --- | --- |
| Human serum 1 | 1×10^-12^ | 1.126×10^-12^ | 5.67 |
| Human serum 2 | 1×10^-12^ | 1.019×10^-12^ | 8.71 |
| Human serum 3 | 1×10^-12^ | 0.998×10^-12^ | 5.53 |
